# Spatial Transcriptomics and snRNA‐seq Expose CAF Niches Orchestrating Dual Stromal‐Immune Barriers in Hepatocellular Carcinoma

**DOI:** 10.1002/advs.202514661

**Published:** 2025-10-07

**Authors:** Yingxue Li, Changxiang Huan, Haoting Sun, Wei Zhang, Zhen Guo, Chuanyu Li, Jia Yao, Zhiqi Zhang, Shizhe Yu, Qiongzhu Dong, Lunxiu Qin, Jinze Li, Lianqun Zhou

**Affiliations:** ^1^ CAS Key Lab of Bio‐Medical Diagnostics Suzhou Institute of Biomedical Engineering and Technology Chinese Academy of Science Suzhou 215163 China; ^2^ School of Biomedical Engineering (Suzhou) Division of Life Sciences and Medicine University of Science and Technology of China Hefei 230026 China; ^3^ Hepatobiliary Surgery Department of General Surgery Huashan Hospital & Cancer Metastasis Institute Fudan University 12 Urumqi Road (M) Shanghai 200040 China

**Keywords:** Cancer‐associated fibroblasts, Hepatocellular carcinoma, Immunosuppressive Microenvironment, single‐nucleus RNA sequencing, Spatial transcriptomics

## Abstract

Hepatocellular carcinoma (HCC) exhibits profound spatial heterogeneity driving therapeutic resistance, while the role of cancer‐associated fibroblasts (CAFs) in orchestrating immunosuppressive niches remains incompletely defined. This study integrates single‐nucleus RNA sequencing (snRNA‐seq) and spatial transcriptomics (stRNA‐seq) to map the cellular and molecular landscape of HCC. snRNA‐seq identifies key cell populations—including fibroblasts, T_NK cells, and endothelial cells—using canonical marker genes. Spatial transcriptomics maps gene expression across tumor regions (core, invasive front, stroma) via the robust cell type decomposition (RCTD) algorithm. Immunofluorescence validates collagen deposition and POSTN spatial distribution, confirming T‐cell exclusion patterns. The analysis identifies hypoxic metabolic myofibroblasts (hmmyCAFs) as central regulators of the tumor microenvironment. hmmyCAFs enrich at the invasive front, forming collagen‐rich barriers that physically exclude CD8⁺ T cells. Simultaneously, they secrete POSTN to suppress immune checkpoint signaling and drive hypoxia‐mediated glycolytic reprogramming of T‐cell metabolism. Clinically, hmmyCAF activity and POSTN expression correlate with reduced progression‐free survival and immunotherapy resistance. This multimodal study defines hmmyCAFs as triple architects of physical immunosuppression, molecular regulation, and metabolic remodeling. By linking collagen remodeling, POSTN‐mediated checkpoint inhibition, and hypoxia‐driven metabolic reprogramming to clinical outcomes, hmmyCAFs and POSTN may serve as potential indicators for evaluating the efficacy of immunotherapy in HCC.

## Graphical abstract

HmmyCAFs may form a triple immunosuppressive niche: possibly secrete ECM (POSTN, etc.) as physical barriers to block CD8⁺ T cells, induce CD8⁺ T exhaustion via those molecules, and use HIF‐1α‐driven metabolism to create acidic, nutrient‐poor microenvironments that suppress T cells.

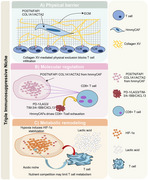

## 1 Introduction

Hepatocellular carcinoma (HCC), the most common histological subtype of liver cancer, is the third‐leading cause of cancer‐related death worldwide.^[^ ^1^ ^]^ Although patients with resectable HCC can be treated with surgical resection or liver transplantation, and patients with advanced unresectable HCC can be treated with sorafenib, these tumors are prone to recurrence after treatment.^[^ ^2^ ^]^ Despite advances in immune checkpoint blockade (ICB) therapy, such as anti‐PD‐1/L1 antibodies, treatment resistance remains a major obstacle, with over 90% of deaths attributed to metastasis or refractory disease. ^[^ ^3^ ^]^A critical barrier to overcoming resistance lies in the profound intratumoral heterogeneity of HCC,^[^ ^4^ ^]^ where spatially and functionally distinct tumor cell subpopulations dynamically interact with components of the tumor microenvironment (TME),^[^ ^5^ ^]^ including immune and stromal cells such as cancer‐associated fibroblasts (CAFs).^[^ ^6^ ^]^ These interactions drive adaptive evolution and therapeutic evasion.

Deciphering this heterogeneity and its crosstalk with the TME is paramount to overcoming these therapeutic challenges. Recent multi‐omics studies have advanced our understanding of HCC heterogeneity.^[^ ^6^, ^7^ ^]^ Integrated analyses of metastatic cohorts have uncovered clonal evolution trajectories, subclone selection pressures, and the pro‐tumorigenic roles of CAFs and B cells in metastasis.^[^ ^8^ ^]^ Large‐scale projects like the China Liver Cancer Atlas (CLCA), which performed deep whole‐genome sequencing on 494 tumors, have delineated population‐specific mutational landscapes and evolutionary patterns.^[^ ^9^ ^]^ Spatially resolved approaches, such as tumor phylogeography, have further resolved spatially blocked heterogeneity by integrating genomic, transcriptomic, and physical coordinates.^[^ ^10^ ^]^ However, these studies predominantly rely on bulk multi‐sample analyses (reporting population averages) or multi‐region sampling with limited spatial resolution, obscuring cellular‐level diversity. Although transformative in other malignancies, single‐cell technologies remain underexploited in HCC research. Consequently, prevailing molecular classifications fail to adequately dissect the interplay between tumor cell‐intrinsic heterogeneity and microenvironmental contributions, or elucidate how their combined action drives metastatic mechanisms and TME‐mediated resistance, leaving critical knowledge gaps. Critically, the central role of CAFs in sculpting an immunosuppressive TME and spatially modulating CD8⁺ T cell distribution and function necessitates deeper exploration at single‐cell resolution and spatial context.​

CAFs are established contributors to the immunosuppressive milieu in HCC ^[^ ^11^ ^]^ However, the precise mechanisms by which they spatially orchestrate immune evasion within specific topographical structures like collagen‐rich exclusion zones and molecularly suppress the functionality of infiltrating CD8⁺ T cells remain poorly defined.^[^ ^4^, ^12^ ^]^ Hypoxia‐driven metabolic reprogramming further compounds this complexity. Yet, constrained by the aforementioned technological limitations in resolution, the precise functional landscape of CAFs within distinct spatial niches, along with their interactions with immune and metabolic networks, remains largely unmapped.^[^ ^13^, ^14^, ^15^ This critical knowledge deficit fundamentally impedes the development of effective CAF‐targeted strategies to overcome ICB resistance. Given the escalating global incidence of HCC, driven largely by the rising prevalence of metabolic dysfunction‐associated steatotic liver disease (MASLD),^[^ ^16^ ^]^ elucidating the core functions of CAFs within the spatially heterogeneous TME is a research imperative with profound clinical urgency.

To overcome these limitations, we integrated single‐nuclear RNA sequencing (snRNA‐seq) and spatial transcriptomics (stRNA‐seq) to systematically map the cellular and spatial heterogeneity landscape of HCC.^[^ ^17^ ^]^ This analysis identified hypoxic metabolic myofibroblasts (hmmyCAFs) as the central orchestrators of the HCC microenvironment. HmmyCAFs dominated the invasive front (Ro/e = 2.7, *p*<0.001), forming peritumoral collagen ribbons. These ribbons established physical CD8⁺ T‐cell exclusion zones via collagen XV; critically, hmmyCAFs proximity intrinsically drove T‐cell dysfunction beyond exclusion. HmmyCAFs concurrently drove immunosuppression via matrix remodeling, oncogenic signaling, hypoxia‐metabolic reprogramming, and multifaceted factor secretion. Clinically, elevated HmmyCAFs /POSTN levels predicted poor progression‐free survival (HR = 0.66, *p* = 0.02) and immunotherapy resistance, linked to reduced TMB (r = ‐0.22, *p* = 2.04e‐05) and suppressed key immune checkpoints (e.g., ADORA2A). Thus, HmmyCAFs‐constructed stromal ribbons are pivotal HCC immune evasion effectors, integrating: 1) physical T‐cell exclusion, 2) POSTN‐driven molecular suppression, and 3) hypoxia immunometabolic disruption. We propose hmmyCAF/POSTN spatial density as a novel biomarker for prognosis and ICB response. Deciphering the immunological microenvironment of HCC offers crucial insights for treating advanced HCC and accelerating therapeutic innovation.

## 2 Results

### 2.1 snRNA‐seq Data Revealed the Cellular Composition of HCC

**Figure 1:**
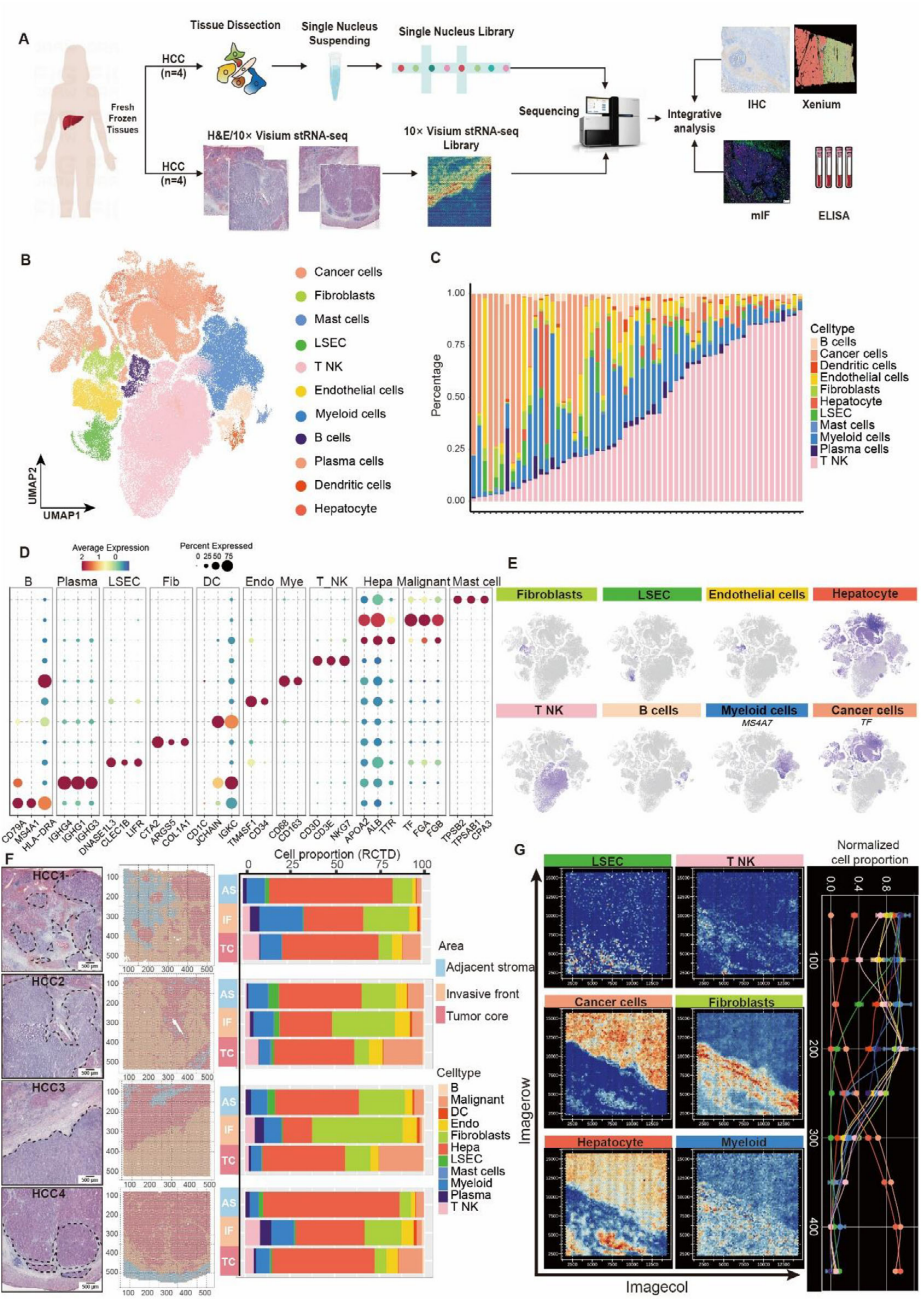
The cellular composition of HCC tissues. A) Workflow of snRNA‐seq and stRNA‐seq experiments applied to HCC tissues. B) UMAP of cells identified from the snRNA‐seq data of four HCC tissues. C) The cell number and proportion of each cell type from each sample. D) Expression of selected marker genes in the major cell types of HCC tissues. Abbreviations: B, B cell; Plasma, plasma cell; LSEC, liver sinusoids endothelial cell; Fib, fibrablast; DC, dendritic cell; Endo, endothelial cell; Mye, myeloid cell; T_NK, a mixed subpopulation of T cell and NK cell; Hepa, hepatocyte; Malignant, malignant cell. E) Expression matrix of cell‐type marker genes in the 11 cell types isolated from HCC tissues. F) Cell‐type proportions across three tissue compartments. Abbreviations: AS, adjacent stroma; IF, invasive front; TC, tumor core. The black dashed line is the tumor‐stromal boundary. Scale bar = 500 µm. G) Spatial distribution of six major cell types within tissue compartments.

To fully characterize the cell composition of the hepatic tissues, we collected HCC samples from one patient with four different parts for snRNA‐seq (**Figure** **1**A). We combined three public datasets and our own test dataset, a total of 22,8045 cells and 43684 genes passed quality control (Figure 1B; Figure S1; Table S1, Supporting Information), from which we identified 11 cell types based on canonical cell markers (Figure 1C; Table S2, Supporting Information), including hepatocyte (3800), fibroblasts (5328), mast cells (689), liver sinusoids endothelial cells (LSEC; 5672), T_NK cells (a mixed subpopulation of T cells and NK cells) (9,7277), endothelial cells (7708), myeloid cells (27 073), B cells (4241), plasma cells (3328), and dendritic cells (DCs) (909). The liver contains two types of cells lining its surface: parenchymal cells are mainly hepatocytes, and heterogeneous non‐parenchymal cells (NPCs) are mainly composed of T_NK cells, myeloid cells, fibroblasts, and LSEC. LSEC, fibroblasts, and endothelial cells are the major cell types present in the liver stroma. LSEC highly expressed CLEC1B, DNASEL3, and LIFR, fibroblasts expressed high levels of COL3A1 in addition to CTA2 and ARGS5, while endothelial cells could be distinguished by high expression of TM4SF1and CD34 (Figure 1D,E). In addition to the structural cells of the liver, diverse immune cell types were also identified, with T_NK cells (CD3D, CD3E, NKG7), B cells (CD79A, MS4A1, HLA−DRA), mast cells (TPSB2, TPSAB1, CPA3), DCs (CD1C, JCHAIN, IGKC), and plasma cells (IGHG4, IGHG1, IGHG3) being the most abundant (Figure 1D,E). In short, the snRNA‐seq data revealed the structural and immune cell composition of the HCC tissues, facilitating downstream spatial transcriptomic analysis with specific cellular gene expression profiles. Unbiased clustering analysis partitioned the whole tissue section into three distinct spatial compartments: Tumor Core, Adjacent Stroma, and Invasive Front (Figure 1F). Given the inherent resolution limitation of Visium technology (55 µm spot^−1^), we employed the robust cell type decomposition (RCTD) algorithm to deconvolute spatial transcriptomic data using single‐cell RNA sequencing as a reference. Cell‐type proportions across compartments were rigorously quantified, revealing fibroblasts and myeloid cells predominantly localized within the invasive front region (Figure 1G). We introduced a set of HCC Xenium data (with single‐cell spatial resolution) to validate the spatial distribution of cells. Results showed that fibroblasts, myeloid cells, LSECs, and T_NK cells were enriched in the non‐tumor core region of HCC (Figure S2, Supporting Information).

### 2.2 Spatial Transcriptomic Characterization of HCC

Spatial information is indispensable for resolving cell–cell interaction networks within tissues, a critical dimension unattainable through snRNA‐seq.^[^ ^18^ ^]^ To overcome this constraint, we used stRNA‐seq to capture in situ gene expression patterns. The stRNA‐seq platform includes capture probes featuring a 16 bp spatial barcode for positional encoding, a 12 bp unique molecular identifier (UMI) for transcript quantification, and a 30 bp poly (T) tail for mRNA hybridization. Liver tissue samples from one HCC patient were collected, with four spatially distinct regions sampled, embedded in OCT compound, and processed into consecutive 10 µm‐thick cryosections. These sections were allocated to stRNA‐seq, hematoxylin and eosin (H&E) histology, and immunohistochemistry (IHC) staining, generating four stRNA‐seq slides. Each capture spot on the stRNA‐seq array spanned 55 µm in diameter, spaced at 100 µm center‐to‐center intervals (Figure S1A, Supporting Information).

**Figure 2:**
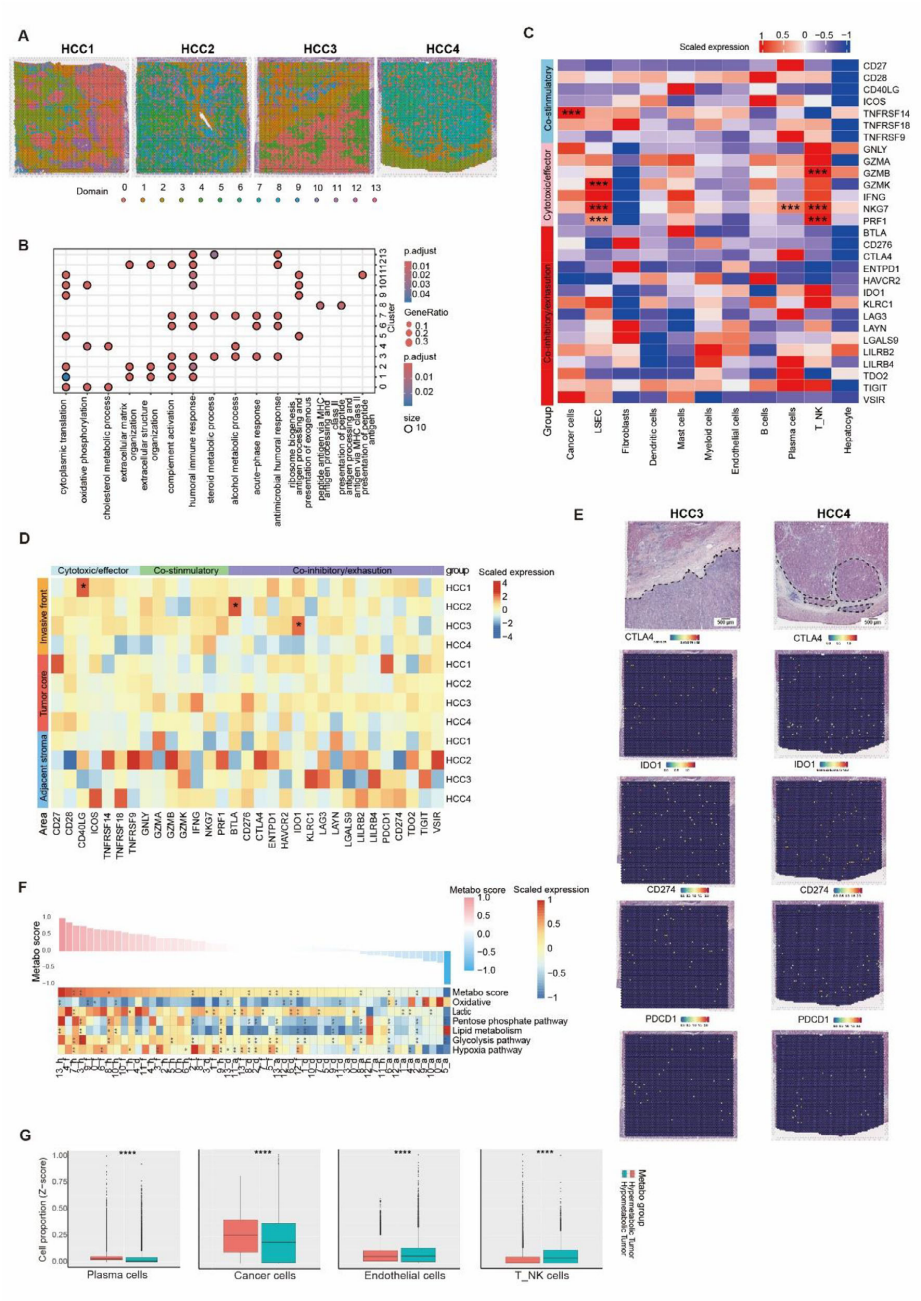
Transcriptomic analysis of the immunity and energy metabolism heterogeneity in HCC. A) The spatial transcriptome divides cell groups. B) GO enrichment of major stRNA‐seq clusters. C) Heatmap showing the expression of gene sets associated with different immune functions (co‐stimulatory, cytotoxic/effector, and co‐inhibitory/exhaustion) in cell types identified by snRNA‐seq data. Abbreviations: LSEC, liver sinusoids endothelial cell; T_NK, a mixed subpopulation of T cell and NK cell. The *p* values were determined by the Wilcoxon signed‐rank test: ^***^ *p <* 0.001. D) Heatmap showing the expression of gene sets associated with different immune functions (co‐stimulatory, cytotoxic/effector, and co‐inhibitory/exhaustion) in stRNA‐seq clusters. The *p*‐values were determined by the Wilcoxon signed‐rank test: ^*^ *p <* 0.05. E) Spatial expression of selected immune checkpoint genes (CTLA4, IDO1, CD274, and PDCD1) in two representative stRNA‐seq samples. The genes tended to be enriched in tumor areas. The black dashed line is the tumor‐stromal boundary. Scale bar = 500 µm. F) Top, metabolic scores corresponding to the tumor areas of the 4 HCC samples. Bottom, heatmap showing the GSVA scores of hypoxias, glycolysis, pentose phosphate, and lipid metabolism pathways for tumor areas from each stRNA‐seq sample. The *p*‐values were determined by the Wilcoxon signed‐rank test: ^**^ *p <* 0.01. G) Violin plots showing abundance of T_NK cells, plasma cells, cancer cells, fibroblasts, and endothelial cells in hyper‐ (n = 10) and hypo‐metabolic (n = 10) tumors. The Y axis shows the GSVA scores for each cell type. The *p* values were determined by the Wilcoxon signed‐rank test: ^****^ *p* < 0.0001.

Spatial clustering via FindClusters function in the Seurat package delineated 14 tissue domains (Figure 2A). Gene Ontology (GO) enrichment analysis revealed pronounced transcriptional/translational activity, proliferative signaling, oxidative phosphorylation, and immune activation within tumor regions compared to adjacent non‐tumor areas. Strikingly, fibroblast‐enriched clusters exhibited upregulated pathways linked to antigen processing and presentation of peptide/polysaccharide antigen via MHC class II and fatty acid metabolic processes, underscoring their metabolic and immunomodulatory roles in the HCC microenvironment (Figure 2B).

### 2.3 Variable Immune Inhibition in HCC

To elucidate the underlying mechanisms of low response rates to PD‐1 blockade and lenvatinib therapy in HCC, we scrutinized the tumor immune landscape.​ The expression profiles of three gene sets with different immune functions, that is, co‐stimulatory, cytotoxic/effector, and co‐inhibitory/exhaustion, were evaluated. At the single‐cell level, co‐stimulatory genes were expressed in cells of both innate and adaptive immunity, especially in CD4+T cells, CD8+T cells, and NK cells (Figure 2C). CD4+T cells highly express CD27, CD28, CD40LG, ICOS, TNFRSF18, TNFRSF14, and TNFRSF9. Overexpression of CD27 within CD4⁺ T cells may suppress anti‐tumor immunity by enhancing regulatory T cell (Treg) function. Although critical for Treg maturation and normal suppressive activity, aberrantly high CD27 levels in Tregs could potentially inhibit effective anti‐tumor immune responses.^[^ ^19^ ^]^ Co‐stimulatory genes demonstrated significant enrichment within invasive front and tumor core regions across spatial transcriptomic datasets (Figure 2D; Table S3, Supporting Information). TNFRSF18 (GITR), in particular, exhibited broad expression within both invasive front and tumor core regions. Notably, its expression was also elevated in epithelial cells of non‐cancerous tissues. snRNA‐seq profiling identified TNFRSF18 expression predominantly within Tregs, NK cells, and T cells (Figure 2C). While linked to Treg‐mediated immunosuppression in tumors, its widespread spatial expression suggests contributions from multiple immune cell populations. Cytotoxic/effector genes (GNLY, GZMA, GZMK, IFNG, NKG7, PRF1) were primarily expressed by T and NK cells (Figure 2C), with notable upregulation in stRNA‐seq‐defined invasive front and tumor core regions (Figure 2D). NK cell‐specific expression was prominent, highlighting their likely role in cytotoxic responses against HCC. Significant expression of canonical immune checkpoint genes CTLA4 and PDCD1 (PD‐1) was not detected within our stRNA‐seq data (Figure 2D), despite robust expression identified by snRNA‐seq (CTLA4 in Tregs; PDCD1 in Tregs, T, and NK cells; Figure 2C). CD274 (PD‐L1), primarily expressed by plasmacytoid DCs, showed tumor/stroma overexpression in only a minor subset of stRNA‐seq samples. While CD276, ENTPD1, IDO1, LGALS9, and VSIR were generally detectable, only IDO1 and LGALS9 consistently exhibited higher expression levels in HCC samples compared to non‐cancerous controls (Figure 2D). These DC‐expressed genes (Figure 2C) are known to dampen cytotoxic T cell activity,^[^ ^20^ ^]^ suggesting their potential viability as alternative immune checkpoint targets over CTLA4 or PD‐L1/PD‐1 for HCC immunotherapy. This hypothesis warrants further investigation. Close examination within individual stRNA‐seq slides revealed substantial spatial heterogeneity in the expression levels of immune‐related genes across distinct tumor regions. When we zoomed in to check the immune genes in the same stRNA‐seq slide, their expression varied greatly between different tumor areas. In sample HCC4, the tumor areas commonly expressed high IDO1, low PD‐L1 and CD274, and very low CTLA4 (Figure 2E). In contrast, the tumor areas commonly expressed high CTLA4, low PD‐L1 and CD274, and very low IDO1 in sample HCC3 (Figure 2E; Figure S3, Supporting Information).

### 2.4 Energy Metabolic Statuses of Tumors were Associated with Different Immune Responses

Energy metabolism represents a hallmark of cancer biology, tightly regulated by oxygen availability and metabolic flux through distinct pathways. Emerging evidence suggests that tumor‐specific metabolic reprogramming dynamically shapes the immune microenvironment, offering novel therapeutic opportunities. To systematically investigate this relationship, we performed Gene Set Variation Analysis (GSVA) across four metabolically relevant pathways: hypoxia, glycolysis, pentose phosphate, and lipid metabolism pathway(Figure 2F; Table S4, Supporting Information). Tumors were stratified into metabolic phenotypes based on composite GSVA scores – the top 20% scoring regions were designated as hypermetabolic clusters, while the bottom 20% constituted hypometabolic clusters (Figure 2F). Hypermetabolic regions exhibited pronounced activation of oxidative phosphorylation (OXPHOS), glycolytic flux, and lactate production, characteristic of the Warburg effect in proliferating tumor populations. Notably, these regions demonstrated concurrent hypoxic signaling and elevated lipid metabolic activity, suggesting compensatory responses to metabolic stress in rapidly growing neoplasms. Distinct immune landscapes between metabolic phenotypes: Hypometabolic zones showed significant enrichment of Endothelial cells and T_NK cells (*p*<0.001), indicative of innate immune activation. Conversely, hypermetabolic regions were infiltrated by immunosuppressive populations, including plasma cells and cancer cells, suggesting compromised adaptive immunity (Figure 2G). Spatial heterogeneity of metabolic states was evident in two cases (HCC4 and HCC1), where adjacent hyper‐ and hypometabolic clusters coexisted within individual tumors (Figure 2G). This intra‐tumoral metabolic compartmentalization may reflect distinct microenvironmental pressures driving evolutionary adaptation, with potential implications for precision immunotherapy strategies targeting metabolic checkpoints.

### 2.5 Characterization of Hypoxic Metabolic Cancer Associated Myofibroblasts (hmmyCAFs) in HCC with snRNA‐seq Data

**Figure 3:**
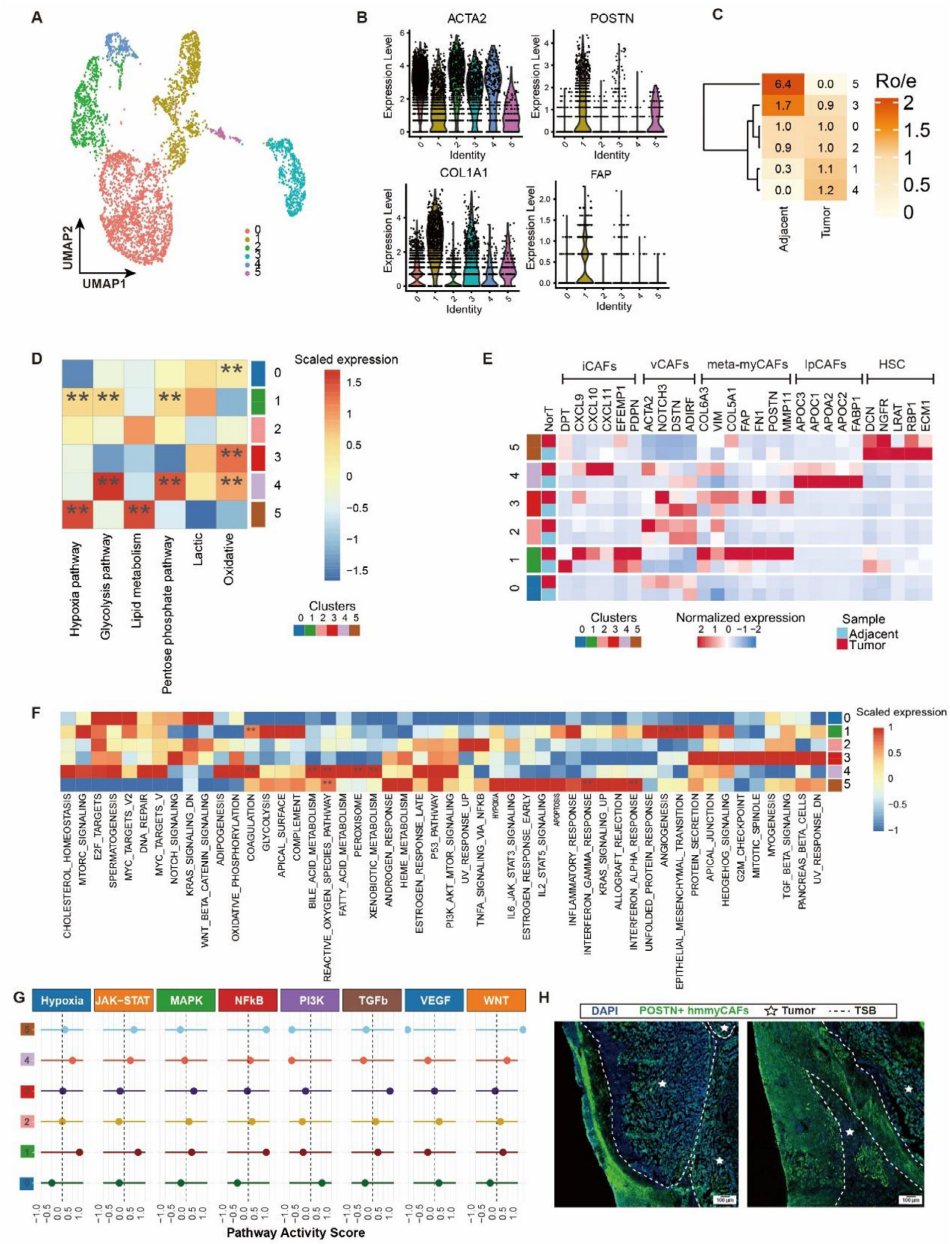
Identification of hypoxic metabolic cancer‐associated myofibroblasts (hmmyCAFs) in HCC. A) UMAP of 4762 CAFs. B) Violin plot showing the expression levels of ACTA2, POSTN, COL1A1, and FAP in the 6 clusters of CAFs. C) Observed‐to‐expected ratio (Ro/e) for fibroblast subclusters across tissue compartments. D) Heatmap showing the expression levels of different subtypes of CAFs in the CAFs subgroup through different metabolic pathways. The *p*‐values were determined by the Wilcoxon signed‐rank test: ^**^ *p <* 0.01. E) Heatmap showing the expression levels of marker genes for different subtypes of CAFs in the subclusters of CAFs. Abbreviations: meCAFs, CAFs with a highly activated metabolic state; apCAFs, antigen‐presenting CAFs; iCAFs, inflammatory CAFs; meta‐myCAFs, metabolic cancer‐associated myofibroblasts; vCAFs, vascular CAFs. F) GSVA results of CAFs (4762 cells). The *p*‐values were determined by the Wilcoxon signed‐rank test: ^**^ *p <* 0.01. G) Signature enrichment analysis of myCAF‐associated signaling pathway. H) IF staining showing the presence of hmmyCAFs in HCC tissues. Tumors can be recognized by the densely packed nuclei stained by DAPI (blue), indicated by white pentacles. POSTN+ hmmyCAFs were stained by green. Abbreviations: TSB, Tumor‐Stromal Boundary. Scale bar = 100 µm.

Spatial analysis of metabolic tumor regions revealed a stromal ribbon‐like architecture peripheral to hypermetabolic zones, prompting snRNA‐seq analysis of fibroblast populations. Across four samples, we identified 4762 CAFs (**Figure** **3**A) exhibiting a CAFs defined by co‐expression of ACTA2, stromal remodeling factors (POSTN, FAP), and collagen regulators (COL1A1) (Figure 3B). Subcluster 1 exhibited pronounced upregulation of extracellular matrix (ECM)‐related genes (COL6A3, COL5A1) in tumor samples compared to normal tissues (Figure 3C). Furthermore, through the analysis of the metabolic pathway activity of the CAF subcluster, it was found that subcluster 1 was highly expressed in pathways such as hypoxia, glycolysis, pentose phosphorylation, and lipid metabolism (Figure 3D; Figure S4, Supporting Information). Based on this profibrotic transcriptional signature and highly metabolic characteristic, we designated this cluster as metabolic myCAFs. To quantify spatial enrichment patterns, we computed the observed‐to‐expected ratio (Ro/e) for fibroblast subclusters across tissue compartments. Metabolic myCAFs demonstrated significant tumor‐specific enrichment (Ro/e = 2.7, *p*  = 3.1 × 10^−5^), exceeding random distribution expectations (Figure 3C). This topographical preference aligns with their role in peritumoral desmoplastic reaction. Functional characterization through MSigDB hallmark analysis (v7.4) demonstrated dual pathway activation in metabolic myCAFs: 1) Stromal programs including UV response suppression, angiogenesis, and EMT regulation; 2) Oncogenic signaling encompassing p53/KRAS pathway modulation, cell cycle control (G2/M checkpoint, E2F targets), and estrogen response (Figure 3E,F). Transcriptomically, these cells maintained collagen biosynthesis machinery (COL1A1/3A1/4A1/5A2/6A3) while aberrantly expressing proliferation markers (TOP2A, MKI67) (Table S5, Supporting Information), suggesting their active role in microenvironment remodeling through combined matrix deposition and self‐renewal capacity. To delineate differentiation‐associated regulatory pathways in metabolic myCAFs, we computed tumor pathway activity scores using the progeny R package (v1.18.0). Among fibroblast subclusters, metabolic myCAFs demonstrated selective enrichment for hypoxia pathway activity (Figure 3G), characterized by coordinated upregulation of hypoxia‐inducible factor targets, finally, we designated this myCAFs as hypoxic metabolic myCAFs (hmmyCAFs). MyCAFs in the invasive front region of the tumor exhibited a significant positive correlation with hypoxic metabolism (Spearman rho = 0.53, *p* < 0.001; Figure S5, Supporting Information). This metabolic reprogramming distinguishes hmmyCAFs from other fibroblast subsets in the tumor‐promoting context. We further verified the expression of POSTN in tissues using immunofluorescence (IF) (Figure 3H).

**Figure 4:**
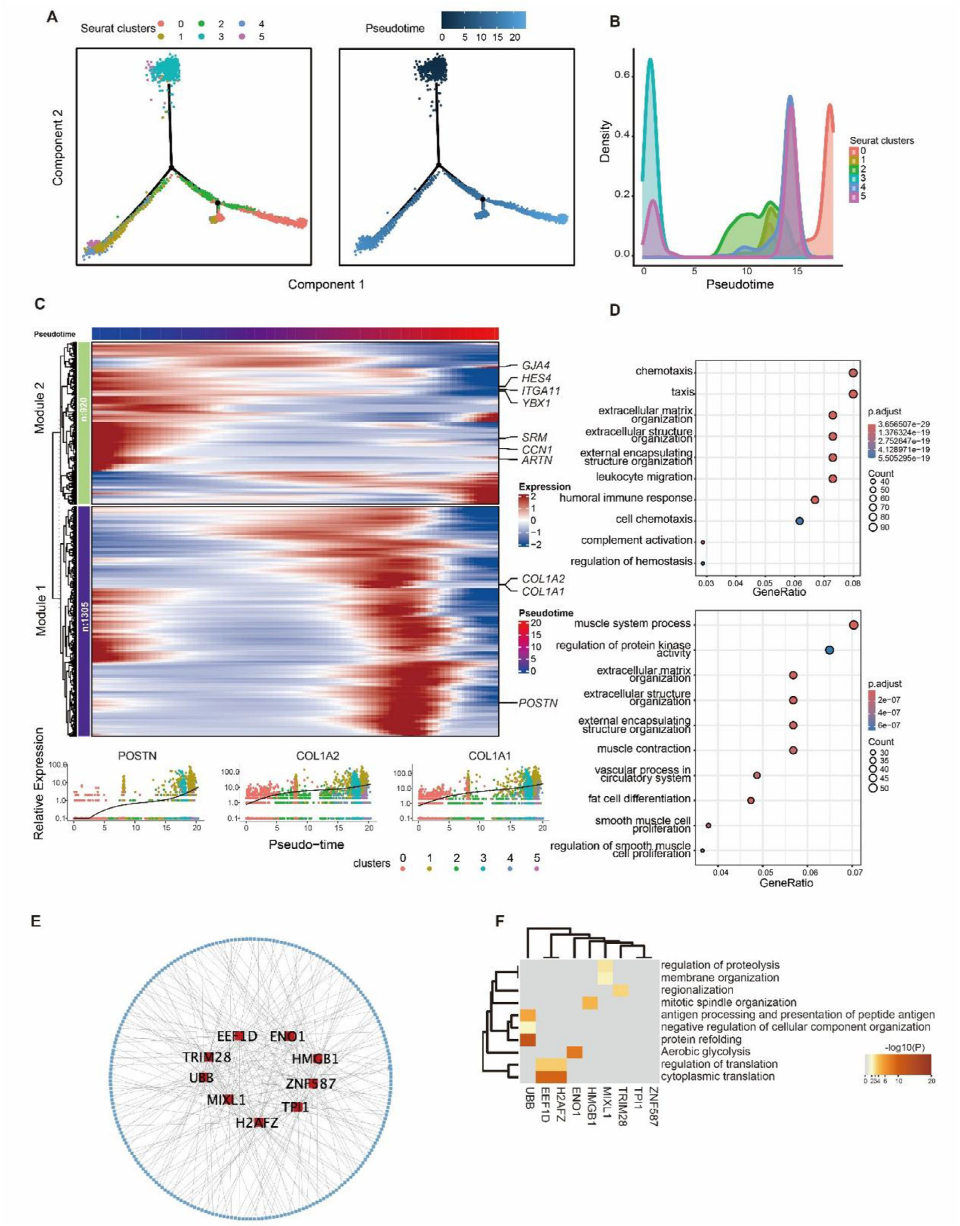
Pseudotime and regulatory network analysis of hmmyCAFs in HCC. A) Pseudotime trajectory of hmmyCAFs (1000 cells) and the other CAFs (3762 cells). B) Density distribution of hmmyCAFs and the other CAFs along the pseudotime trajectory. C) Upper, DEGs between hmmyCAFs and the other CAFs with pseudotime variation; Down, The pseudo‐time relative expression level variation of POSTN, COL1A2, and COL1A1 in different clusters. D) GO enrichment of the gene modules with pesudotime variation. E) Regulatory network of hmmyCAFs. Nine genes in red are regulatory factors (TFs) with over 30 target genes. F) GO enrichment of the target genes of the 9 TFs in hmmyCAFs.

**Figure 5:**
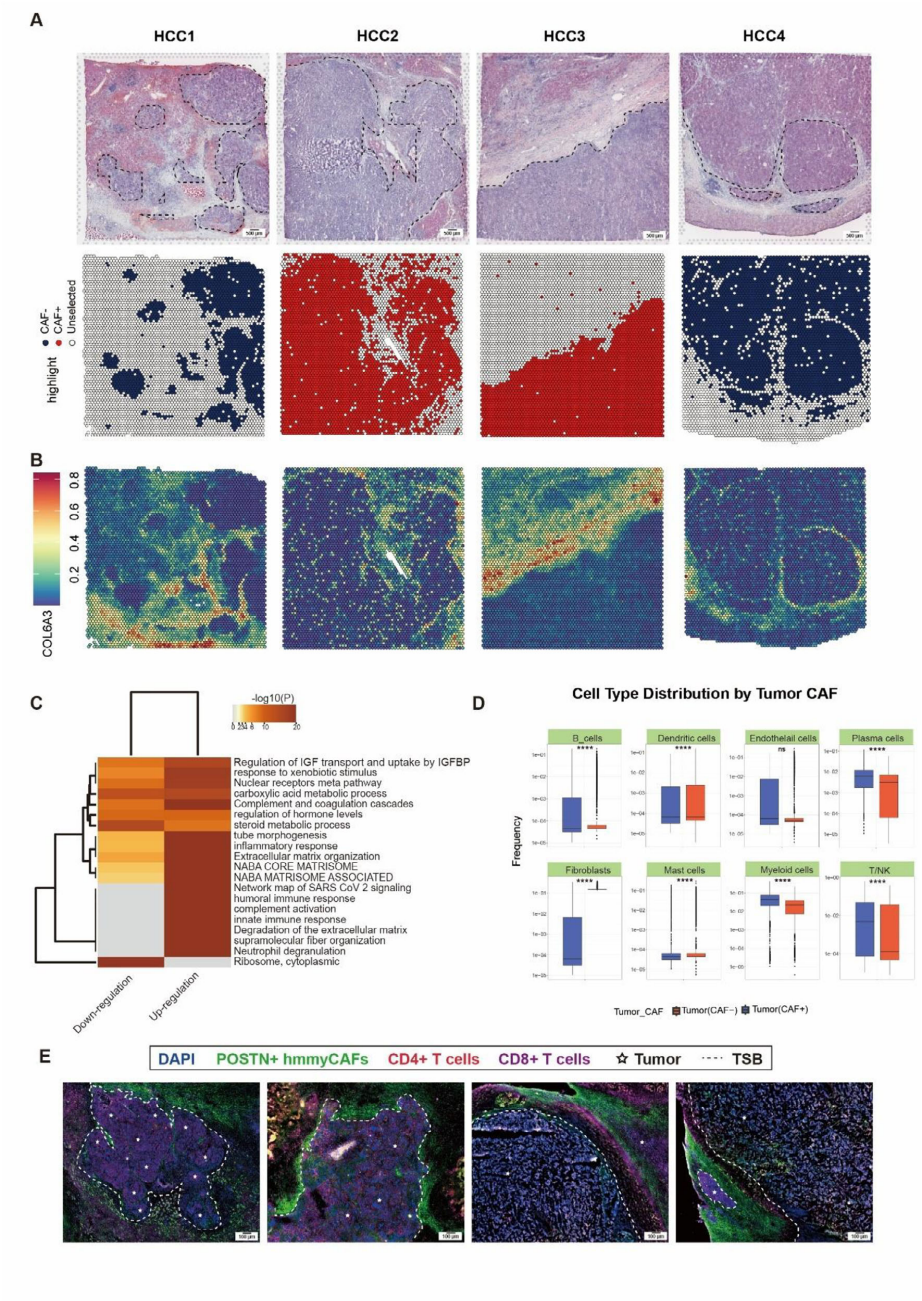
Spatial and functional characterization of hmmyCAFs in HCC. A) Spatially projected hmmyCAFs in representative StRNA‐seq slides. The projected area was determined based on the RCTD cell proportion of hmmyCAFs, POSTN expression pattern, and the IHC staining results of POSTN (see Figure S6B, C, Supporting Information). The black dashed line is the tumor‐stromal boundary. Scale bar = 500 µm. B) Density distribution of hmmyCAFs along the pseudotime trajectory. C) Heatmap enriched GO terms for up‐ and down‐regulated the hmmyCAF+ tumors and hmmyCAF– tumors. D) Box plot showing the abundance of hmmyCAF+ and hmmyCAF– tumors in different cells. The *p* values were determined by Student's t test: ns, not significant; ^*^ *p* < 0.05; ^**^ *p* < 0.01; ^***^ *p* < 0.001; ^****^ *p* < 0.0001. E) mIF staining showing the aggregation of CD4+ and CD8+ immune cells in the stroma adjacent to tumors enclosed by hmmyCAFs. Tumors can be recognized by the densely packed nuclei stained by DAPI (blue), indicated by white pentacles. CD4+ T cells were stained by red and CD8+ T cells were stained by violet. POSTN+ hmmyCAFs were stained by green. Abbreviations: TSB, Tumor‐Stromal Boundary. Scale bar = 100 µm.

Pseudotime analysis of hmmyCAFs and other fibroblasts revealed that hmmyCAFs were positioned at one end of the trajectory (**Figure** **4**A,B). A total of 2225 genes (*p* < 0.05) associated with the pseudotime trajectory of hmmyCAFs were identified (Table S6, Supporting Information),^[^ ^21^ ^]^ which could be divided into two modules. **Figure** **5**C displays the top 100 genes with pseudotime variation. Genes in Modules 1 and 2 exhibited upregulation in hmmyCAFs, such as COL1A1, COL1A2, ITGA11, GJA4, SRM, CCN1, and POSTN, which served as components and markers of myCAFs^[^ ^8^, ^22^ ^]^. Concurrently, genes promoting tumor growth, including HES4, YBX1, and ARTN, were also upregulated in hmmyCAFs. Modules 1 is primarily linked to chemotaxis, humoral immune response, and cell chemotaxis (Figure 4D, top).^[^ ^23^, ^24^, ^25^ ^]^ In contrast, genes in Module 2 showed a downregulation trend in hmmyCAFs (Figure 4C), such as SRM, CCN1, and ARTN. Longitudinal expression dynamics along the fibroblast pseudotime trajectory identified POSTN, COL1A1, and COL1A2 as pivotal regulators driving differentiation into hmmyCAFs (Figure 4C). This module was further associated with multiple cancer‐related signaling pathways, including the muscle system process and regulation of protein kinase activity (Figure 4D, bottom). Regulatory network analysis of hmmyCAFs with GENIE3 revealed 8441 regulatory pairs (Table S7, Supporting Information, weight > 0.05). Among these, 9 transcription factors (TFs) regulated more than 30 genes (Figure 4E), including EEF1D, ENO1, TRIM28, HMGB1, UBB, ZNF587, MIXL1, TPI1, and H2AFZ. These TFs not only participated in protein refolding and cytoplasmic translation but also governed critical tumorigenesis pathways such as aerobic glycolysis, regulation of proteolysis, membrane organization, regionalization, mitotic spindle organization, negative regulation of cellular component organization, antigen processing and presentation of peptide antigen, and regulation of translation pathway (Figure 4F). These characteristics underscore a pivotal role of hmmyCAFs in HCC progression.^[^ ^26^, ^27^ ^]^

### 2.6 The hmmyCAFs Might Facilitate the Growth and Metastasis of HCC from Diverse Aspects

To locate the spatial distribution of hmmyCAFs in StRNA‐seq chips, we adopted the RCTD analysis approach developed by Dylan M. Cable et al. to integrate snRNA‐seq and StRNA‐seq data.^[^ ^28^ ^]^ Briefly, this method calculated the overlapping degree of the expression levels of cell type‐specific genes identified by snRNA‐seq data and the area‐specific genes characterized by StRNA‐seq data. The results showed that hmmyCAFs were enriched around some tumor areas in 2 out of the 4 stRNA‐seq slides (Figure 5A). The presence of hmmyCAFs in HCC was further confirmed by IHC staining of POSTN using serial tissue sections from the same samples (Figure S6C, Supporting Information). Notably, not all tumor areas were surrounded by hmmyCAFs, making us curious about the biological differences associated with the presence of these cells.

To comprehensively elucidate the biological functions of hmmyCAFs in HCC, we classified tumor regions into two categories based on the spatial distribution density of hmmyCAFs in the transcriptomic data: hmmyCAF‐high tumor regions and hmmyCAF‐low tumor regions (Figure 5A). The existence of hmmyCAFs was jointly determined by the RCTD approach and POSTN expression pattern, as previously mentioned. We then used the differentially expressed genes between hmmyCAF+ and hmmyCAF– tumor areas of the four samples to perform GO enrichment analysis (Figure 5B,C; Table S8, Supporting Information). The results showed that the hmmyCAF+ tumors were more active in humoral immune response, complement activation, and inflammatory response complement activation than hmmyCAF– tumors (Figure 5C). Meanwhile, Ribosome, cytoplasmic, and steroid metabolic processes were down‐regulated in hmmyCAF+ tumors (Figure 5C). These observations coincided with the immune and metabolic heterogeneity of HCC (Figure 2F). These results indicate that the presence of hmmyCAFs may support tumor progression from different aspects.

Next, we calculated the gene module expression scores of immune gene sets for the tumor bins to evaluate the immune cell abundance. The results showed significantly reduced numbers of B cells, Mast cells, and DCs in hmmyCAF+ tumors (Figure 5D), indicating that hmmyCAFs might act as a physical barrier to prevent the infiltration of pro‐immunity cells into tumor areas. To confirm this, we conducted mIF to measure the spatial distribution of hmmyCAFs, CD4+ cells, and CD8+ cells using POSTN, CD4, and CD8 as markers, respectively. The results showed the accumulation of CD4+ and CD8+ cells outside the tumors enclosed by hmmyCAFs, especially CD4+ cells (Figure 5E). In addition, we cocultured hmmyCAF and T cells and found that a significant upregulation of genes associated with T cell exhaustion (PDCD1, LAG3, HAVCR2, TNFRSF9, ENTPD1, and CXCL13), suggesting that hmmyCAFs may inhibit pro‐immunogenic cell functions (Figure S7, Supporting Information).

### 2.7 The Presence of hmmyCAFs was Associated with Poorer Clinical Status of HCC

**Figure 6:**
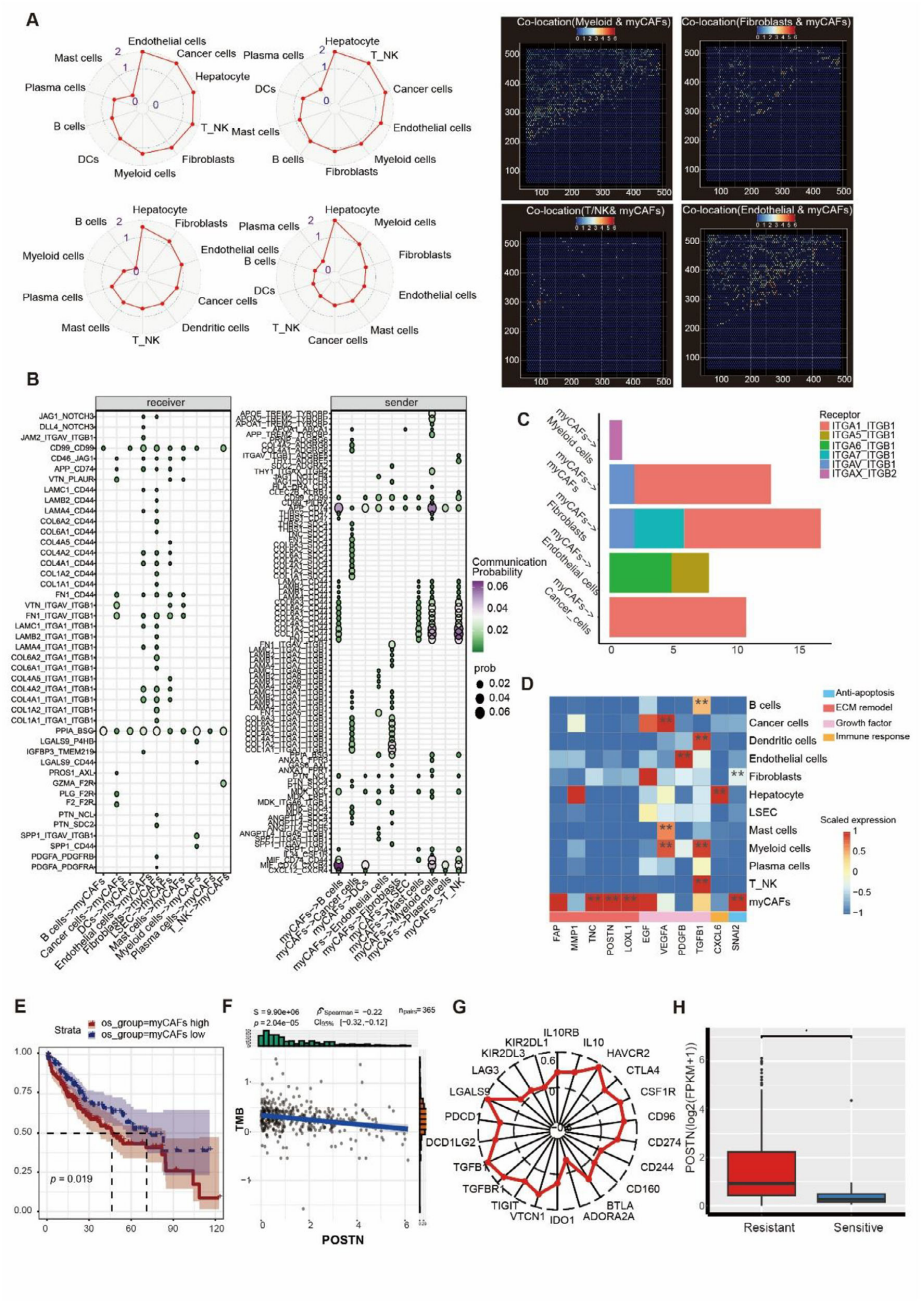
Functional analysis of hmmyCAF in HCC. A) Left, Radar plot illustrating co‐localization of hmmyCAFs with other cell types; Right, Spatial location heatmap illustrating co‐localization of hmmyCAF with other cell types. Abbreviations: DCs, dendritic cells; B) Ligand‐receptor communication network between hmmyCAFs and different cells predicted by snRNA‐seq data. The cell–cell communication probability was estimated by integrating gene expression with prior knowledge of the interactions between signaling ligands, receptors, and their cofactors. Left, expression bubble map of top receptors regulated by hmmyCAFs in different cell types. Right, bubble map of the top predicted receptors expressed by hmmyCAFs. LSECs, liver sinusoidal endothelial cells. C) Bar plot showing the integrin types involved in potential communications between hmmyCAFs and the other cell types. D) Expression heatmap of gene sets related to functions of hmmyCAFs in tumor development in snRNA‐seq data. The *p*‐values were determined by the Wilcoxon signed‐rank test: ^**^ *p <* 0.01. E) The progression‐free survival probabilities of patients with HCC were estimated based on the signature scores of the marker gene set for hmmyCAFs. The analysis was conducted using a 365 HCC dataset from TCGA. F) The relationships between POSTN expression and TMB scores in the TCGA dataset (n = 365). G) The relationships between POSTN expression and common immune genes associated with immunotherapy in the TCGA dataset (n = 365). H) Differential expression of POSTN in immunotherapy‐sensitive (*n* = 10) and resistant (*n* = 242) groups. Expression data presented as mean ± SEM, the *p* values were determined by the Wilcoxon signed‐rank test: ^*^ *p <* 0.05.

Cell–cell communication within the tumor microenvironment predominantly occurs through direct physical contact. Utilizing the mistyR package (v1.6.0), we quantified dominant cellular interactors of hmmyCAFs, identifying endothelial cells and myeloid cells as primary contact partners (**Figure** **6**A). These cell types collectively establish a spatially organized invasive front niche that orchestrates multiple pro‐tumorigenic mechanisms. These stromal cells demonstrated particularly strong involvement in ECM remodeling through elevated expression of collagen genes (COL1A1/2, COL4A1/2, COL6A1‐3), fibronectin (FN1), and multiple laminin isoforms (LAMA2‐5, LAMB1‐3, LAMC1/3). Notably, hmmyCAFs exhibited cell‐type‐specific engagement of integrin heterodimers for microenvironmental crosstalk (Figure 6B). Tumor cell interactions preferentially involved α2β1, α3β1, and αvβ8 integrin combinations, while vascular compartment communication (endothelial cells, circulating endothelial cells, smooth muscle cells) utilized α9β1, α6β1, and α1β1 configurations. Immune modulation of T_NK cells occurred through distinct α1β1‐mediated binding. This receptor specialization suggests hmmyCAFs employ molecular adaptation to fulfill context‐dependent roles in tumor‐stromal interactions. The identification of these ligand‐receptor pairs highlights two complementary mechanisms: direct cell–cell contact through membrane‐bound receptors, and matrix‐mediated signaling via secreted glycoproteins. Integrins, as heterodimeric transmembrane receptors coordinating both adhesion and signaling, appear central to hmmyCAF‐mediated regulation of cellular migration and microenvironment organization.

Mechanistically, hmmyCAFs appear to orchestrate tumor microenvironment modulation through dual barrier and signaling functions (Figure 6C,D). These cells leverage F11R/JAM1‐mediated intercellular junctions through JAM3 interactions with cancer and stromal cells, potentially establishing physical exclusion zones that limit immunocyte infiltration. Complementing this barrier mechanism, hmmyCAFs secrete a specialized matrix repertoire containing thrombospondins (THBS1/2) and tenascin C (TNC), which engage CD44 receptors, α3β1 integrins, and syndecan‐1 (SDC1) to mediate cross‐talk with diverse cellular populations, including plasma cells and smooth muscle cells.

The extracellular remodeling capacity of hmmyCAFs is further amplified by their overexpression of multifunctional tissue‐reshaping effectors (Figure 5C). This molecular repertoire encompasses: 1) structural organizers (POSTN, LOXL1) enabling matrix crosslinking; 2) proteolytic regulators (FAP, MMP1) facilitating matrix degradation; and 3) multifunctional glycoproteins (TNC) that bridge mechanical and signaling functions. Such coordinated expression of matrix‐modifying enzymes and structural components positions hmmyCAFs as central architects of tumor‐associated ECM dynamics.

Beyond ECM modulation, hmmyCAFs deploy a multifaceted secretome to potentiate malignant progression through stemness regulation and proliferative signaling (Figure 6B,D). The pleiotropic factor SEMA3C maintains cancer stem cell populations while driving angiogenesis and invasive processes.^[^ ^29^ ^]^ POSTN demonstrates dual signaling activation through integrin αvβ3‐mediated Akt/PKB survival pathways and Wnt/β‐catenin cascades that sustain tumor expansion.^[^ ^30^, ^31^ ^]^ CXCL6, while canonically immunomodulatory, paradoxically accelerates neoplastic growth and metastatic dissemination in HCC.^[^ ^32^ ^]^

The molecular arsenal extends to include: 1) SNAI2‐mediated apoptosis resistance through transcriptional reprogramming; ^[^ ^33^, ^34^ ^]^ 2) Conventional mitogenic drivers (TGFB1, EGF, VEGFA) amplifying proliferative signaling; and 3) Immune subversion mechanisms via LSD1‐mediated interferon suppression and Wnt5a‐induced immunosuppressive niche formation.^[^ ^35^, ^36^ ^]^ Notably, Wnt5a secretion establishes immunosuppressive niches conducive to metastatic spread^[^ ^37^ ^]^ (Figure 6B). This coordinated expression of stemness‐enhancing factors, survival signals, and immune‐modulating components reveals hmmyCAFs as metabolic architects of TME optimization. Their dual capacity to reinforce stromal support networks while actively dismantling anti‐tumor immunity underscores a central role in malignant evolution.

To investigate the tumor‐promoting capacity of hmmyCAFs, we conducted survival analysis on a HCC cohort from The Cancer Genome Atlas (TCGA) containing 365 cases. Gene Set Variation Analysis (GSVA) quantification of hmmyCAF activity was performed using a signature panel (ACTA2, POSTN, COL1A1, FAP), revealing that elevated hmmyCAF activity demonstrated significant association with reduced progression‐free survival (HR = 0.66, 95% CI = 0.47‐0.94, *p* = 0.0202; Figure 6E). Subsequently, we examined the relationship between hmmyCAF infiltration and established immunotherapy response predictors: tumor mutation burden (TMB), microsatellite instability (MSI), and immune checkpoint expression profiles (PD‐1/PD‐L1, CTLA‐4, TIM‐3). Patients with a higher level of TMB, MSI, or immune inhibitory gene expression tended to be more sensitive to immunotherapy. ^[^ ^38^ ^]^ Strikingly, POSTN expression (a key hmmyCAF marker) exhibited significant inverse correlations with TMB scores (Spearman's rho = ‐0.22, *p* = 2.04e‐05; Figure 6F), suggesting potential mechanistic links between hmmyCAF abundance and immunotherapy resistance in HCC.

Notably, POSTN expression demonstrated inverse correlations with key immune checkpoint regulators (ADORA2A, CD160, CD244; Figure 6G), while exhibiting positive associations with VEGFR2, a dual‐function mediator of both angiogenic signaling and metastasis‐associated immunosuppression.^[^ ^39^ ^]^ To evaluate clinical implications, we applied ImmuCellAI algorithms to stratify TCGA‐HCC patients (N = 365) by predicted immunotherapy responsiveness. Strikingly, POSTN levels were significantly attenuated in the immunotherapy‐sensitive subgroup (n = 10) compared to resistant counterparts (n = 411, *p*<0.005, Figure 6H). By measuring the POSTN levels in paired serum samples from advanced HCC patients before and after PD‐1 immunotherapy, it was found that POSTN expression was lower before treatment and significantly increased after immunotherapy (Figure S8, Supporting Information). Mechanistically, these findings suggest hmmyCAF‐derived POSTN may orchestrate immune evasion through two complementary pathways: 1) Downregulation of T‐cell inhibitory checkpoints, and 2) VEGF receptor‐mediated angiogenesis that establishes immunosuppressive vascular niches. The convergence of elevated POSTN with immunotherapy resistance patterns (reduced TMB, diminished checkpoint expression) supports its potential as a biomarker for immune checkpoint blockade (ICB) non‐responsiveness. Therapeutic implications emerge from this multi‐omics evidence: Combinatorial strategies pairing angiogenesis inhibitors with ICB may circumvent hmmyCAF‐mediated immunosuppression, potentially restoring anti‐tumor immunity in HCC patients with stromal‐rich microenvironments.

**Figure 7:**
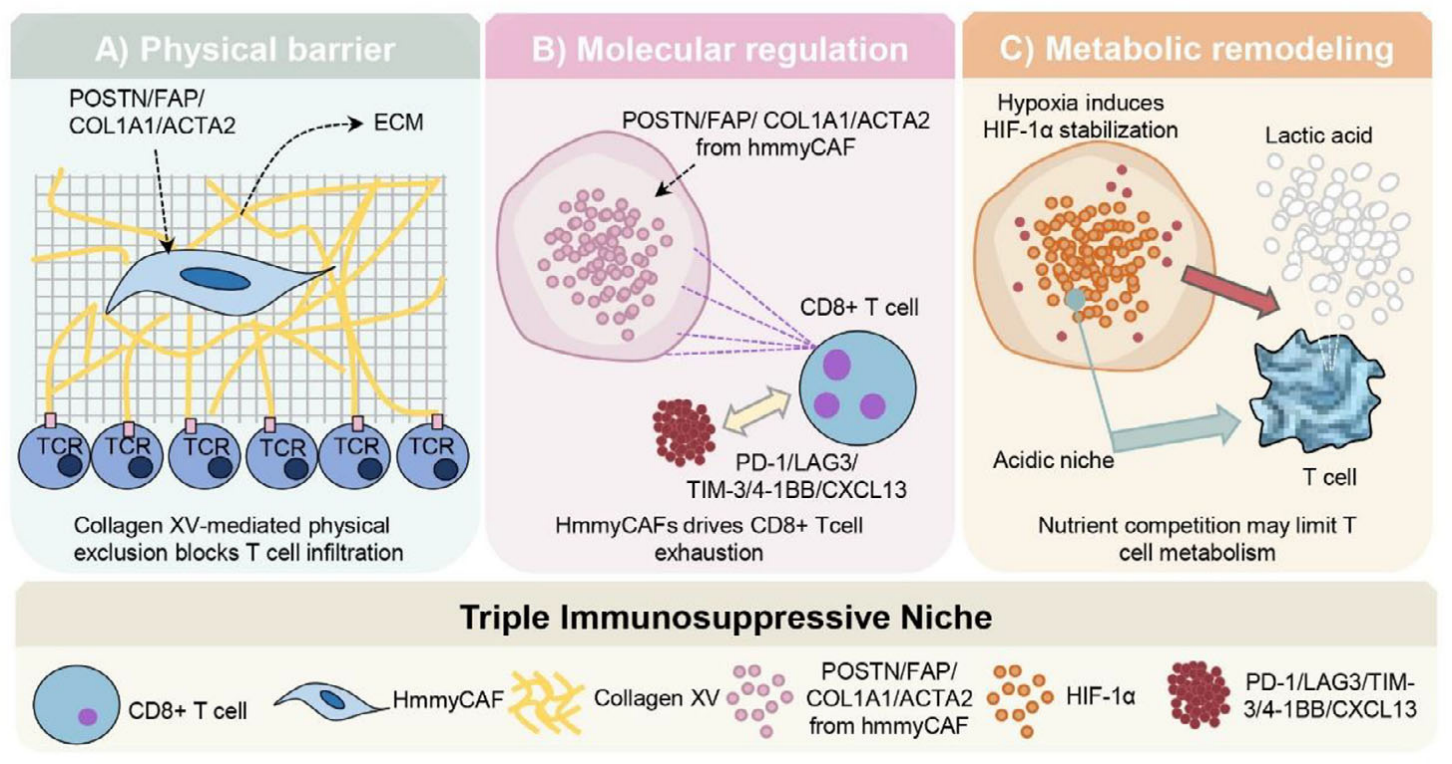
Mechanisms underlying the triple immunossuppressive niche orchestrated by hmmyCAFs through physical barrier formation, molecular regulation, and metabolic remodeling. A) Physical barrier. HmmyCAFs secrete ECM components, including POSTN, FAP, COL1A1, and ACTA2, to assemble a dense ECM. Collagen XV mediates physical exclusion, restricting the infiltration of CD8⁺ T cells (identified by T cell receptor (TCR) labeling) into the tumor microenvironment. B) Molecular regulation. HmmyCAFs‐derived POSTN, FAP, COL1A1, and ACTA2 act on CD8⁺ T cells, inducing the expression of exhaustion‐associated markers (PD‐1, LAG3, TIM‐3, 4‐1BB, and CXCL13). This process drives CD8⁺ T cell exhaustion, a state of functional impairment characterized by reduced cytokine production and cytotoxic activity. C) Metabolic regulation. Hypoxia stabilizes HIF‐1α (Hypoxia‐inducible factor 1‐alpha) in HmmyCAFs, triggering metabolic reprogramming. This results in lactic acid secretion, establishing an acidic niche that suppresses T cell metabolism. Additionally, nutrient competition between HmmyCAFs and CD8⁺ T cells further limits T cell access to essential metabolites, exacerbating T cell dysfunction.

Collectively, our results indicated that hmmyCAFs are a crucial component of the TME of HCC and drive a triple immunosuppressive niche via three pathways. Physically, secreted ECM components (POSTN/FAP/COL1A1/ACTA2) form a dense matrix; collagen XV restricts CD8⁺ T cell infiltration. Molecularly, these components induce T cell exhaustion by upregulating exhaustion markers (PD‐1/LAG3/TIM‐3/4‐1BB/CXCL13). Metabolically, hypoxia – stabilized HIF – 1α in HmmyCAFs triggers lactic acid secretion (acidic niche) and nutrient competition, limiting T cell access to metabolites and impairing function (**Figure** **7**).

## 3 Discussion

Our snRNA‐seq delineates the stromal composition of HCC at single‐cell resolution, identifying fibroblasts as a core component of the HCC stroma. Together with LSECs, fibroblasts, and endothelial cells  constitute the primary stromal cell populations in HCC (Figure 1). Spatial deconvolution further demonstrates selective enrichment of fibroblasts and myeloid cells within the invasive front (Figure 1F), consistent with their roles in barrier formation and immunomodulation. Notably, LSECs exhibit elevated expression of immunoregulatory genes (CLEC1B, DNASE1L3), suggesting their contribution to immune tolerance through ECM remodeling. These findings address a critical gap in prior bulk‐tissue analyses, ^[^ ^40^, ^41^ ^]^ providing unprecedented insight into the spatial architecture of the TME. Specifically, the selective enrichment of fibroblasts at the invasive front underscores their potential role as key drivers of tumor invasion (e.g., by breaching the basement membrane and facilitating tissue penetration), while their immune regulatory functions—collaborating with LSECs (which mediate immune tolerance through ECM remodeling)—collectively establish the immunosuppressive microenvironment of HCC. This single‐cell‐level characterization significantly advances our understanding of the spatial and functional heterogeneity of the HCC TME.

Spatial transcriptomics uncovers pronounced intra‐tumoral metabolic heterogeneity: hypermetabolic regions (enriched for oxidative phosphorylation and glycolytic pathways) recruit cytotoxic T_NK cells (*p* < 0.001), whereas hypometabolic niches (characterized by hypoxia and lipid metabolism) accumulate immunosuppressive plasma cells and malignant cells (Figure 2F,G). This metabolic‐immune codistribution elucidates regional immunotherapy resistance—hypoxic zones simultaneously exclude effector lymphocytes while enriching immunosuppressive populations, creating “immune deserts.” This mechanistic understanding highlights the therapeutic potential of targeting metabolic checkpoints (e.g., hypoxia‐responsive nanotherapeutics) to overcome current barriers in immunotherapy.

Central to these mechanisms are hypoxic metabolic myCAFs (hmmyCAFs), which act as architects of spatially organized immunosuppressive niches. Co‐expressing ACTA2, POSTN, FAP, and COL1A1 (Figure 3), hmmyCAFs are enriched at the invasive front (Ro/e = 2.7, *p*<0.001; Figure 3C). They establish dual barriers: collagen XV‐dependent physical exclusion (segregating CD8+ T cells; Figure 5E) and POSTN‐driven molecular suppression (secreting POSTN, SEMA3C, CXCL6 to inhibit T cells). Spatial correlation analysis reveals hmmyCAFs also drive T‐cell dysfunction via integrin signaling (α2β1/αvβ8 with tumor cells; α1β1 with T_NK cells) and activation of the POSTN‐αvβ3‐Akt/Wnt axis (Figure 6B,D), creating Wnt5a‐enriched niches that facilitate immune evasion.^[^ ^42^ ^]^ Hypoxia‐mediated metabolic reprogramming (Figure 3G) and transcription factor‐driven differentiation (Figure 4G,H) epigenetically enforce their specialization.

Clinical variables further modulate hmmyCAF function in immunotherapy resistance.^[^ ^43^, ^44^ ^]^ In advanced HCC (III‐IV), hmmyCAFs spatially expand from the invasive front to the tumor core (Ro/e = 2.7 to 4.1, *p*<0.001; Figure 3C), driven by TGF‐β activation. This proliferation enhances immune suppression: collagen XV mediates CD8+ T cell exclusion (reduced infiltration; Figure 5E). Pre‐fibrotic changes (e.g., sinusoidal capillarization) synergize with hmmyCAF‐secreted collagen XV (Figure 5E) to reinforce the physical barrier function. In cirrhosis, myeloid cell enrichment (Figure 2G) further drives immune suppression by secreting IL‐10 and TGF‐β, which suppress DC antigen presentation. This combination may form an “immunosuppressive loop,” ultimately reducing ICB response rates. Collectively, these variables modulate hmmyCAF spatial distribution, metabolism, and secretion, collectively shaping the TME's immune suppressive capacity and determining immunotherapy response.

The clinical significance of this stromal‐immune axis is underscored by robust correlations between hmmyCAFs activity and adverse outcomes: elevated POSTN expression predicts inferior progression‐free survival (HR = 0.66, *p* = 0.02; Figure 6E) and drives immunotherapy resistance through TMB suppression (Spearman's r = ‐0.22, *p* = 2.04e‐05; Figure 6F) and downregulation of critical immune checkpoint molecules (ADORA2A, CD160; Figure 6G), with significantly lower levels observed in therapy‐responsive patients (*p*<0.005; Figure 6H). Consequently, POSTN spatial density emerges as a dual biomarker for both prognostication and rational therapeutic stratification, guiding combinations of anti‐angiogenic agents with immune checkpoint blockade to overcome microenvironment‐mediated resistance, particularly in the context of rising MASLD‐driven HCC incidence.^[^ ^45^, ^46^ ^]^

We further evaluate emerging therapeutic strategies targeting hmmyCAFs and POSTN, leveraging preclinical and early‐phase clinical data on CAF‐targeting agents. Fibroblast Activation Protein (FAP), a key marker of activated CAFs, including hmmyCAFs, regulates their pro‐tumorigenic functions. Preclinical data support FAP inhibition as a viable strategy. OMTX705,^[^ ^47^ ^]^ a small‐molecule FAP inhibitor, reduces CAF proliferation/collagen secretion in pancreatic models, reversing T cell exclusion via ECM disruption, a mechanism relevant to hmmyCAFs, which rely on collagen XV (Figure 5E) for physical T cell barriers.

TGF‐β drives hmmyCAF to immunosuppressive phenotypes via enhanced signaling (r = 0.41, *p* = 0.008; Figure 3D). TGF‐β blockers (e.g., galunisertib) reduce TGF‐β‐induced POSTN and CAF immune suppression, downregulating hmmyCAF POSTN and sensitize T cells to ICB.^[^ ^47^ ^]^ Lorvotuzumab (anti‐TGF‐β) enhances ICB efficacy by reducing CAF activation, disrupting hypoxia adaptation, and alleviating lactate‐mediated T cell dysfunction.^[^ ^48^ ^]^ Combining anti‐angiogenics (e.g., VEGF inhibitors) may boost efficacy. Bevacizumab (anti‐VEGF) normalizes tumor vasculature to enhance T cell infiltration; ^[^ ^49^, ^50^ ^]^ paired with galunisertib, it may suppress hmmyCAF TGF‐β signaling, disrupting collagen XV barriers and POSTN‐checkpoint interactions. These strategies (targeting FAP, TGF‐β, or anti‐angiogenics) may disrupt hmmyCAF‐mediated immunotherapy resistance by addressing their activation, signaling, and function. Future studies should validate these combinations in stratified HCC cohorts to optimize selection and translate insights.

This study establishes hmmyCAFs as pivotal regulators of HCC immune evasion, with their spatial organization and dual mechanisms (physical barriers + molecular suppression) positioning them as high‐value therapeutic targets. Targeting strategies can be prioritized across three levels: 1. Block the POSTN signaling axis: hmmyCAFs specifically overexpress POSTN (Figure 3H), which induces T cell exhaustion via the αvβ3‐Akt/Wnt pathway (Figure 6D). Preclinical validation in ovarian cancer supports this approach: anti‐POSTN neutralizing antibodies (e.g., mAb 10A6) inhibit tumor growth/metastasis;^[^ ^51^ ^]^ 2. Exploit metabolic vulnerability: The hypoxic metabolic signature of hmmyCAFs (GSVA‐enriched hypoxia/glycolysis pathways; Figure 3G) renders them susceptible to LDHA inhibitors. This strategy has successfully depleted hypoxic CAFs and enhanced immunotherapy in lung adenocarcinoma;^[^ ^52^ ^]^ 3. Develop combinatorial regimens: Given the association between hmmyCAF abundance and immunotherapy resistance (high POSTN correlates with PFS HR = 0.66, *p* = 0.02; Figure 6E), combining FAP‐targeted CAF depletion with anti‐PD‐1 therapy could simultaneously disrupt physical barriers and molecular inhibition—mirroring pancreatic cancer models where this approach doubled survival.^[^ ^53^ ^]^

However, clinical translation faces two key challenges:1. Targeting specificity: Avoiding damage to non‐hmmyCAF subpopulations (e.g., quiescent fibroblasts) involved in tissue repair; 2. Subtype plasticity: hmmyCAFs may evade therapy via phenotypic switching^[^ ^54^, ^55^ ^]^ (e.g., FAP downregulation, metabolic reprogramming).

Future studies must validate hmmyCAF‐specific markers in immune‐competent HCC organoids and explore patient stratification based on spatial hmmyCAF density to optimize “stromal reprogramming‐immune activation” synergies. While our findings offer novel insights into hmmyCAFs in HCC immunotherapy resistance, we acknowledge limitations. First, our core single‐cell/spatial analyses used a single patient's tumor (four regions), capturing intratumoral heterogeneity (Figure 3C) but potentially underrepresenting interpatient variability. To mitigate this, we validated key findings via public cohorts (TCGA, GEO, GSA‐Human), showing consistent POSTN‐immune evasion associations across HCC populations (Figure 6E). Nevertheless, prospective validation in larger, multi‐center cohorts (diverse etiologies/stages) remains critical to establish clinical relevance. Future studies will validate findings in independent, well‐annotated cohorts using multi‐omics to clarify clinical‐hmmyCAF‐immunotherapy relationships, enabling precise biomarkers and stratification strategies for HCC immunotherapies.

## 4 Experimental Section

### Patients and Samples

Tumor tissues were collected from 1 patient aged 38–69 years from the Department of Hepatobiliary Surgery, Affiliated Cancer Hospital of Fudan University. Freshly collected samples collected at four locations in different regions were used for snRNA‐seq and stRNA‐seq. In addition, paired plasma samples from 11 HCC patients before and after PD‐1 treatment were collected at the same hospital for ELISA testing of POSTN to evaluate the efficacy of immunotherapy.

### Ethical Statement

The study was approved by the Ethics Advisory Committee of Huashan Hospital, Shanghai Medical College, Fudan University (KY2020‐1228, Nov. 2020). Written informed consents were obtained from each of the involved individuals.

### Cell Lines and Culture

Human LX‐2 cells (Cat# GNHu58) and Jurkat cells (Cat# SCSP‐513) were purchased from the National Collection of Authenticated Cell Cultures (China). These cell lines were subjected to short tandem repeat (STR) testing at the time of purchase. The STR testing results were provided by the vendor and third‐party testing agencies, ensuring the authenticity of the cell lines. All cell lines were cultured in RPMI‐1640 (Thermo Fisher Scientific, USA) supplemented with 10% fetal bovine serum (FBS, HyClone, USA) and 1% penicillin/streptomycin (Gibco, USA) at 37 °C with 5% CO_2_.

### Coculture

A non‐contact transwell coculture system was established using 0.4 µm pore size Transwell inserts (Corning, USA). 1 × 10⁶ LX‐2 cells were seeded in the lower chamber, while 0.7–0.9 × 10⁶ cells were placed in the upper chamber. The study included two groups: (i) a coculture group (TGF‐β‐preconditioned LX‐2 cells cocultured with Jurkat cells) and (ii) a control group (Jurkat cells cultured alone). For the coculture group, LX‐2 cells in the lower chamber were first treated with 20 µg mL^−1^ recombinant TGF‐β (Novoprotein, Shanghai, China) for 24 h. Subsequently, the conditioned medium in the lower chamber was replaced with fresh RPMI‐1640 (Thermo Fisher Scientific, USA), and Jurkat cells were seeded into the upper chamber to initiate coculture.

### ELISA Test

Plasma samples (≈10 µL) were analyzed for POSTN protein levels using a commercial ELISA kit (Yfxbio Biotech, Nanjing, China) following the manufacturer's instructions. Absorbance was measured using an Infinite F50 microplate reader (TECAN, Switzerland). All remaining plasma samples were stored at ‐80 °C for future analysis.

### Quantitative real‐time PCR

Total RNA was extracted from cultured cells using RNA‐easy Isolation Reagent (Vazyme, Nanjing, China). RNA quality and quantity were assessed using a NanoDrop 2000 spectrophotometer (Thermo Fisher Scientific, USA). Complementary DNA (cDNA) was synthesized using a commercially available kit (TransGen Biotech, Beijing, China) following the manufacturers’ instructions. Real‐time quantitative PCR was performed on a ABI Q5 Real‐Time System (Thermo Fisher Scientific, USA) using Taq Pro Universal SYBR qPCR Master Mix (Vazyme, Nanjing, China) to quantify mRNA levels. Gene expression levels were normalized to the housekeeping gene GAPDH, and relative quantification was calculated using the 2^−^^ΔΔ^Ct method. Primer sequences for all target genes are listed in Table S9 (Supporting Information).

### Data Collection

To ensure reproducibility and comprehensively map the HCC tumor microenvironment, multiple publicly available single‐cell transcriptomic datasets were integrated. Specifically, the GSE149614 (n = 21) and GSE156625 (n = 78) datasets were obtained from the Gene Expression Omnibus (GEO) repository (https://www.ncbi.nlm.nih.gov/geo/). The CRA002308 dataset was retrieved from the Genome Sequence Archive for Human (GSA‐Human). For large‐scale cohort validation, processed RNA‐seq data and clinicopathological metadata from TCGA‐LIHC were publicly retrieved through TCGA data portal (https://portal.gdc.cancer.gov/). All datasets underwent rigorous quality control and normalization to enable cross‐platform comparability. This integrative approach leveraged spatial, single‐cell, and bulk transcriptomic resources to dissect stromal‐immune crosstalk and malignant cell heterogeneity in HCC.

### Tissue Preparation for Spatial Transcriptomic Experiment

The collected tumor tissues were rinsed with cold PBS, and embedded with pre‐cooled OCT (Sakura, USA) in dry ice for 10 min and stored in a refrigerator at −80 °C. The OCT embedded tissue blocks were trimmed to an appropriate size and sliced in a −20 °C freezer (Thermo, USA). Three to four serial cryosections of 10 µm thickness were cut from the OCT‐embedded samples for H&E staining, stRNA‐seq library preparation, and IF staining. Brightfield images of the H&E samples were obtained using a RX50 microscope scanner (Sunny Optical, China) for histopathological assessment.

### Single‐Nucleus RNA Sequencing

The remaining tissues of stRNA‐seq were used for snRNA‐seq. Nuclei isolation and permeabilization were performed under the guidance of the Chromium Next GEM Single‐Cell Multiome ATAC + Gene Expression User Guide (CG000338). snRNA‐seq libraries were prepared using Chromium Single Cell 3ʹ Reagent Kits v3 (10× Genomics, USA) according to the manufacturer's instructions. Briefly, high‐quality sequencing data were obtained after a series of experimental procedures, including cell counting and quality control, gel beads‐in‐emulsion (GEMs) generation and barcoding, post‐GEM‐RT cleanup, cDNA amplification, gene expression library construction, and NovaSeq platform (Illumina, USA) sequencing.

### Quality Control of RNA Obtained from OCT‐Embedded Samples

Briefly, 10 µm thick sections (≈10–15 cryosections) were cut from each OCT‐embedded sample for total RNA extraction using the RNeasyMini Kit (Qiagen, USA) according to the manufacturer's protocol. RNA integrity number (RIN) was determined using a 2100 Bioanalyzer (Agilent, USA). Only samples with RIN ≥ 7 were qualified for the transcriptomic study. All samples had an RIN of 7–10.

### stRNA‐seq Library Preparation and Sequencing

The spatial transcriptomic RNA library was constructed using stRNA‐seq capture chips (10× genomics, USA) with a size of 0.65 cm^2^. The capture spots were 220 nm in diameter, with a center‐to‐center distance of 100 µm. Each stRNA‐seq capture probe contained a 16 bp coordinate identity barcode, a 12 bp molecular identity barcode, and a 30 bp poly T tail for in situ mRNA hybridization. A cryosection of 10 µm thickness cut from OCT‐embedded tissue was quickly placed on the chip, incubated at 37 °C for 1 min, and then fixed in pre‐cooled methanol at −20 °C for 30 min. The fixed tissue sections were stained with Qubit ssDNA dye (Thermo Fisher, USA) to check the tissue integrity before fluorescent imaging. The tissue sections were then permeabilized using 0.1% pepsin (Sigma, USA) in 0.01 mol L^−1^ HCl buffer, incubated at 37 °C for 14 min, and then washed with 0.1×SSC. RNA released from the permeabilized tissue was reverse‐transcribed for 1 h at 42 °C. The tissue sections were then digested with a tissue removal buffer at 42 °C for 30 min. The cDNA‐containing chip was then subjected to cDNA‐release enzyme treatment at 55 °C. The released cDNA was further amplified using a cDNA HIFI PCR mix (KAPA). Approximately 20 ng of cDNA was fragmented to 400–600 bp, amplified for 13 cycles, and purified to generate a DNA library, which was sequenced with PE150 (Illumina, USA).

### IHC Staining

IHC staining of POSTN was performed according to the manufacturer's protocol. The frozen sections were dried at room temperature, placed in an oven at 37 °C for 10–20 min, fixed with 4% paraformaldehyde for 20 min, and washed thrice with PBS (pH = 7.4) for 5 min. The antigens were then repaired with EDTA (pH 9.0), and endogenous peroxidase was blocked with 3% hydrogen peroxide. The slides were blocked with 3% BSA (G5001‐100 g, Servicebio) at room temperature for 30 min and then incubated with POSTN (19899‐1‐AP, Proteintech, 1:200) at 4 °C overnight. Finally, the frozen slices were subjected to secondary antibody blocking, DAB staining, nuclear restaining, and dehydration. The protein expression levels of POSTN were evaluated under a microscope by professional pathologists.

### mIF Staining

mIF staining was performed to simultaneously detect 1) the location of hmmyCAFs (POSTN), 2) the spatial relationship between the tumor, hmmyCAFs, and immunocytes (CD4+ and CD8+). Briefly, tissue sections from FF samples were fixed by formaldehyde and dehydrated. The sections were then incubated with primary antibodies. The details of the primary antibodies used are as follows: POSTN (19899‐1‐AP, Proteintech, 1:200) and CD8 (66868‐1‐IG, Proteintech, 1:200). Then the sections were incubated with secondary antibodies and stained with DAPI (C0060, Solarbio, Beijing). The details of the secondary antibodies used are as follows: CoraLite647 goat anti‐mouse IgG antibody (SA00014‐10, Proteintech, 1:200), Alexa Fluor 488 goat anti‐rabbit IgG antibody (A11008, Invitrogen, 1:200), and eFluor 570 CD4 antibody (41‐2444‐80, Invitrogen, 20 µg mL^−1^). Fluorescence images were obtained using the Olympus’ SLIDEVIEW VS200 slide scanner (Japan) and were visualized using Olyvia Viewer.

### Quality Control and Gene Expression Quantification of snRNA‐seq Data

The raw single‐cell RNA sequencing data (Fastq files) were processed using the CellRanger toolkit (v9.0) for alignment and quantification against the human reference genome GRCh38. Potential empty droplets with low RNA content were identified using the dropletUtils R package (v1.10.3), which evaluates whether the RNA content of each barcode significantly exceeds background noise levels (FDR < 0.01 threshold for authentic cell retention). Subsequent quality control involved three filtering criteria: 1) Exclusion of barcodes with UMI counts < 30000 per library; 2) Retention of cells containing 500–6000 detected genes; 3) Removal of cells where mitochondrial gene content exceeded 50% of total transcripts. Doublet detection was performed using the doubletFinder function in the scran R package (v1.18.7). Briefly, artificial doublets were computationally generated assuming a default doublet formation rate of 25%. The proportion of artificial nearest neighbors (pANN) was calculated based on principal component analysis (PCA) distance matrices derived from the top 40 principal components and 3000 highly variable genes. Shared nearest neighbor (SNN) graphs were constructed using the FindNeighbors function, followed by Louvain clustering via FindClusters. The final doublet removal threshold incorporated both sample‐specific doublet rates (calculated as 7.5×10^−^⁶ per cell) and homotypic doublet proportions estimated through modelHomotypic. High pANN‐value cells were systematically excluded to ensure dataset purity.

### Data Analysis of Spatial Transcriptomcs

The raw spatial transcriptomic sequencing data and corresponding image files were processed using SpaceRanger (v3.0.1), with alignment and quantification performed against the GRCh38 reference genome. Image files were subsequently aligned with tissue microarray coordinates to enable spatial mapping of gene expression matrices. Downstream analyses utilized the Seurat object constructed through the Load10X_Spatial function, integrating expression matrices with spatially registered images. Quality control criteria for spatial transcriptomic data included: 1) Spot filtering: Retained spots containing >200 detected genes; 2) Gene filtering: Removed genes expressed in fewer than 3 spots. This workflow ensured robust spatial resolution while eliminating low‐information features. Using the Seurat package, the gene matrix and cell segmentation matrix of Xenium data were imported. The Xenium data was selected with a customized 474‐gene panel pre‐designed by 10x Genomics. Cells with no detected genes were removed.

### Cell Type Clustering and Annotation

The integrated dataset underwent downstream analysis in Seurat (v4.4) to identify major cell populations. Batch effects arising from sample sources, dataset variability, and tissue types (tumor vs normal) were mitigated using the RunHarmony function. Following SCTransform‐based normalization of 3000 highly variable genes (HVGs), dimensionality reduction was performed via principal component analysis (PCA) with the top 40 principal components selected for subsequent analyses. Cell clustering was achieved through shared nearest neighbor (SNN) graph construction (FindNeighbors) and Louvain community detection (FindClusters, resolution = 0.6). Eleven major cell types were annotated using canonical marker genes: B cells (CD79A, MS4A1), Fibroblasts (ACTA2, RGS5, COL1A1), Myeloid cells (CD14, CD68, CD163, S100A8), DCs (IL3RA, IRF7, GZMB, CD4), Plasma cells (MZB1, IGHG1, IGLL5), Endothelial cells (VWF, CD34), Hepatic sinusoidal endothelial cells (DNASE1L3, CLEC1B, CLEC4G, CLEC4M), T_NK cells (CD3D, CD3E, CD3G, NKG7, IL7R), and Mast cells (TPSAB1). Hepatocytes and tumor cells, sharing developmental origins in HCC, were grouped based on ALB and APOA2 expression. Malignant cells were discriminated from non‐malignant counterparts through genome‐wide copy‐number variation (CNV) profiling using inferCNV (v1.3.3). Cellular heterogeneity was visualized via uniform manifold approximation and projection (UMAP) in two‐dimensional space (RunUMAP function).

### Cell Clusters Deconvolution

The Visium V2 platform provides a spatial resolution of 55 µm, allowing individual barcodes to capture transcripts from multiple cell types. To resolve cellular composition within each spatial barcode, deconvolution analysis was performed using the RCTD algorithm in the spacexr package (v2.0.0), integrating single‐cell transcriptomic data as a reference. Given that the average cell diameter (≈10 µm) was substantially smaller than the spot size, single 55‐µm spatial spots likely encompass heterogeneous cell populations. Accordingly, RCTD was executed in “full mode” without constraints on the number of permitted cell types per spot. Normalized cellular proportions were stored in the meta.data slot of the Seurat object, enabling downstream spatial visualization and quantitative analysis of cell‐type distributions. This approach rigorously accounts for transcriptional heterogeneity inherent to the Visium platform's technical resolution. Xenium specializes in in situ gene expression analysis at single‐cell and even subcellular resolution. Therefore, Xenium employs the doublet mode in the RCTD package. First_type was selected to annotate each cell.

### Identification of Differentially Expressed Genes (DEGs) and Cell‐Type Marker Genes

To systematically identify DEGs and spatial marker genes across single‐cell and spatial transcriptomic datasets, the FindMarkers and FindAllMarkers functions in Seurat (v4.4) were employed. A non‐parametric Wilcoxon rank‐sum test was applied as the default statistical framework. Resulting *p*‐values underwent multiple‐testing correction using the Bonferroni method. DEGs and marker genes were defined by stringent thresholds: average log2‐fold change (avg_log2FC) > 1 and adjusted *p*‐value < 0.05. This dual‐criterion approach ensured robust identification of transcriptionally distinct features while minimizing false discovery rates.

### Tumor Metabolic Profiling

To investigate regional metabolic heterogeneity within tumors, five tumor‐associated metabolic pathways were extracted from the MSigDB database (https://www.gsea‐msigdb.org/gsea/msigdb/index.jsp): hypoxia pathway,^[^ ^56^ ^]^ glycolysis pathway,^[^ ^57^ ^]^ pentose phosphate pathway,^[^ ^58^ ^]^ and lipid metabolism pathway.^[^ ^59^ ^]^ Metabolic activity scores were calculated using the AddModuleScore function in Seurat. Briefly, background genes were sorted by their mean expression levels across all spots and partitioned into 25 expression bins. For each bin, 100 genes were randomly selected to generate a reference gene set. Metabolic scores for each pathway were computed by comparing the aggregated expression of pathway‐specific genes against this randomized background. These scores, stored in the metadata, enabled spatial mapping of metabolic activity variations across tumor regions, providing insights into microenvironment‐driven metabolic adaptations.

### Tumor Activity Pathway Score

To identify key dysregulated pathway nodes across fibroblast subpopulations, pathway activity scores were computed using the progeny R package (v1.17.0). A human‐specific pre‐trained model was employed to infer activity levels of cancer‐relevant pathways. For each pathway, the top 200 core genes with minimal pathway‐specific *p*‐values were selected to generate pathway activity signatures. Activity scores were calculated for each fibroblast subpopulation using the following workflow: 1) Pathway activity matrices were scaled and centered via ScaleData in Seurat, implementing Z‐score normalization (mean = 0, SD = 1) per pathway; 2) Biological replicates were aggregated to compute mean pathway activities;

3) Subpopulation‐specific pathway dysregulation was quantified by comparing Z‐scores against reference cell types. This approach enabled robust quantification of oncogenic signaling pathway activation (e.g., TGF‐β, WNT, MAPK) within context‐specific fibroblast states.

### Pseudotime Analysis

To delineate developmental pathways and critical transition points among fibroblast subtypes, pseudotemporal ordering was performed using Monocle (v2). Noise reduction was first applied by excluding genes with a mean expression < 1 across cells. The top 3000 highly variable genes (HVGs) from fibroblast subclusters were retained, followed by selection of trajectory‐defining genes showing significant differential expression (adjusted *p*‐value < 0.01). Dimensionality reduction was implemented via the reduceDimension function with the DDRTree algorithm, retaining the first two components for pseudotemporal trajectory visualization. Gene expression dynamics along pseudotime were modeled using the differentialGeneTest function, which employs generalized additive models (GAMs) to identify genes with expression levels significantly correlated (p < 0.05) with progression along the inferred trajectory. Candidate genes were filtered based on: 1) expression > 0 in ≥10 cells; 2) inclusion in the trajectory‐defining gene set. Hierarchical clustering of pseudotime‐dependent genes (via the heatmap package) revealed two distinct co‐expression modules (Module 1 and Module 2), highlighting bifurcation points and transcriptional programs governing fibroblast subtype specification. This dual‐module architecture suggests a branching differentiation model where microenvironmental cues drive subtype divergence through sequential activation of these gene networks.

### Signature Enrichment Analysis of Fibroblasts with snRNA‐seq Data

To investigate pathway‐level heterogeneity among fibroblast subpopulations, comprehensive signature enrichment analysis was performed using the Molecular Signatures Database (MSigDB v7.4). Specifically, hallmark gene sets (e.g., HALLMARK_EPITHELIAL_MESENCHYMAL_TRANSITION) and curated pathway sets (e.g., KEGG_ECM_RECEPTOR_INTERACTION from the C2 collection) were selected to capture and quantify biological processes associated with fibroblast activation, fibrogenesis, and immunomodulation. Pathway activity scores were computed for each cellular subcluster using the AddModuleScore function in Seurat. This algorithm compared target gene sets (HALLMARK pathways) against computationally derived background gene sets (randomly partitioned via 50 control gene bins), generating signature scores that represent normalized pathway activation levels per subpopulation.

### Regulatory Network Analysis of hmmyCAFs

Standard transcription factor (TF) annotations were retrieved from the PySCENIC official repository (https://resources.aertslab.org/). TFs with non‐zero expression levels were retained for downstream analysis. A Random Forest (RF) regression model was implemented to quantify the regulatory impact of each TF on target genes, measured by Variable Importance (VI) scores. The model was configured with 50 decision trees, and potential target genes were filtered based on VI thresholds ^**^> 0.05^**^. TF‐target gene pairs were submitted to the STRING database (https://string‐db.org/) to construct protein‐protein interaction (PPI) networks, retaining interactions with combined confidence scores > 0.4. GO enrichment analysis for network modules was performed using Metascape (v3.5.2023) under default parameters (significance threshold: *p* < 0.01, minimum overlap = 3, enrichment factor > 1.5).

### Cell–Cell Communication

To understand the communication network between hmmyCAFs and other cell types, cell–cell communication was conducted using CellChat with the snRNA‐seq data to obtain the ligand‐receptor pairs regulated by hmmyCAFs (Figure 6A).^[^ ^60^ ^]^ Normalized gene expression matrices and cell‐type annotations were imported into CellChat, with ligand‐receptor pair databases interrogated across three interaction modalities: Secreted Signaling, ECM‐Receptor, and Cell‐Cell Contact. Gene filtering criteria included: 1) Ligand or receptor genes were retained if expressed in ≥10% of cells within a subpopulation (trimmed mean threshold: 0.1); 2) Wilcoxon rank‐sum tests identified genes significantly overexpressed (*p* < 0.05) in specific cell clusters compared to all other cells. A putative hmmyCAF‐driven interaction was defined when: 1) the ligand exhibited significant overexpression in hmmyCAFs (sender population); 2) the cognate receptor showed significant overexpression in the receiver population. This dual‐threshold framework ensured identification of biologically plausible communication axes while minimizing false‐positive signals. Interaction strengths were quantified via CellChat's probabilistic framework, integrating expression magnitudes and pathway‐specific communication probabilities.

### Prognostic Analysis of hmmyCAFs with TCGA Data

Normalized gene expression profiles and clinical metadata from HCC cohorts were retrieved from the TCGA database. Patients were stratified into high‐hmmyCAF (POSTN expression ≥ cohort mean) and low‐hmmyCAF (POSTN expression < cohort mean) groups using POSTN, a validated marker gene for myofibroblastic cancer‐associated fibroblasts. All survival analyses were conducted in R (v4.2.1) using the survival package (v3.4‐0). Key analytical steps included: 1) Computation of hazard ratios (HRs) with 95% confidence intervals (95% CIs) through multivariate Cox proportional‐hazards regression models; 2) Generation of Kaplan‐Meier survival curves via the survfit function; 3) Statistical comparison of survival distributions using two‐sided log‐rank tests.

### Estimating the Effects of hmmyCAFs on Immunotherapy in HCC

To explore the impact of hmmyCAFs on immunotherapy in HCC, the RNA‐seq data of 439 patients with HCC were downloaded from HCC. The Maftools package was used to generate the TMB score of each patient. The MSI scores were determined by Li et al. with MSIsensor. ImmuCellAI was used to predict the response rate of ICB therapy based on the gene expression matrix of the 439 patients. The relationships between POSTN expression and TMB score, MSI score, or immune gene expression were estimated by Spearman's correlation coefficients.

### Statistical Analysis

Statistical significance was determined by the Kruskal–Wallis test and Wilcoxon's rank‐sum test with the Benjamini–Hochberg method for multiple comparison correction. Spearman rank correlation coefficients were calculated using the cor.test function in the base R package. Significance levels were denoted as follows: ns, not significant; ^*^p < 0.05; ^**^p < 0.01; ^***^p < 0.001; ^****^p < 0.0001. All analyses employed two‐tailed tests to ensure rigorous evaluation of associations.

## Conflict of Interest

The authors declare no conflict of interest.

## Author Contributions

Y.L., C.H., and H.S. contributed equally to this work and are co‐first authors. Y.L., C.H., J.L., and L.Z. designed this study. W.Z., Z.G., C.L., J.Y., Z.Z., H.S., Q.D, L.Q., J.L., and L.Z. supervised this study. Y.L., C.H., and H.S. conducted methodology. S.Y. and H.S. coordinated the sample collection. Y.L. conducted snRNA‐seq, stRNA‐seq, H&E, mIF and IHC experiments. W.Z., Z.G., C.L., J.Y., Z.Z., Q.D, L.Q., J.L., and L.Z. advice on experiments. C.H. conducted bioinformatic analysis. Y.L. and C.H. wrote the original manuscript. W.Z., Z.G., C.L., J.Y., Z.Z., H.S., Q.D, L.Q., J.L., and L.Z. reviewed and polished the manuscript.

## Supporting information



Supporting Information

Supplemental Table 1

## Data Availability

The data that support the findings of this study are available from the corresponding author upon reasonable request.
