# Supplementary material for: Spatial Transcriptomics and snRNA‐seq Expose CAF Niches Orchestrating Dual Stromal‐Immune Barriers in Hepatocellular Carcinoma
Source: Adv Sci (Weinh). 2025 Oct 7;12(48):e14661. doi: 10.1002/advs.202514661 (PMC12752642; doi:10.1002/advs.202514661)
Supplement: Supplementary file 1 — Supporting Information [file ADVS-12-e14661-s001.docx]

Supporting Information

**Spatial Transcriptomics and snRNA-seq Expose CAF Niches Orchestrating Dual Stromal-Immune Barriers in Hepatocellular Carcinoma**

Yingxue Li#, Changxiang Huan#, Haoting Sun#, Wei Zhang, Zhen Guo, Chuanyu Li, Jia Yao, Zhiqi Zhang, Shizhe Yu, Qiongzhu Dong, Lunxiu Qin*, Jinze Li*, Lianqun Zhou*

Y. Li, W. Zhang, Z. Guo, C. Li, J. Yao, Z. Zhang, J. Li, L. Zhou

CAS Key Lab of Bio-Medical Diagnostics, Suzhou Institute of Biomedical Engineering and Technology, Chinese Academy of Science, Suzhou, 215163, China

C. Huan, W. Zhang, Z. Guo, C. Li, J. Yao, Z. Zhang, J. Li, L. Zhou

School of Biomedical Engineering (Suzhou), Division of Life Sciences and Medicine, University of Science and Technology of China, Hefei, 230026, China.

S. Yu, H. Sun, Z. Dong, X. Lun

Hepatobiliary Surgery, Department of General Surgery, Huashan Hospital & Cancer Metastasis Institute, Fudan University, 12 Urumqi Road (M), Shanghai, 200040, China.

#: The authors contributed equally to this work and are co-ﬁrst authors.

*: Co-corresponding authors.

**1. Supporting Tables (see Excel files)**

Table S1. snRNA-seq data statistics.

Table S2. Marker genes for the annotation of cell types in HCC.

Table S3. Expression matrix of immune genes in stRNA-seq areas.

Table S4. GSVA gene sets used to characterize the metabolic statuses of the ST tumor areas.

Table S5. DEGs for fibroblasts, hmmyCAFs, and cancer cells.

Table S6. DEGs between hmmyCAFs and the other fibroblasts.

Table S7. Regulatory genes and their targets identified in hmmyCAFs (weight > 0.05).

Table S8. DEGs for hmmyCAF+ and hmmyCAF- tumors.

Table S9. Primer sequence information for quantitative real-time PCR.

**2. Supporting Figures**

**
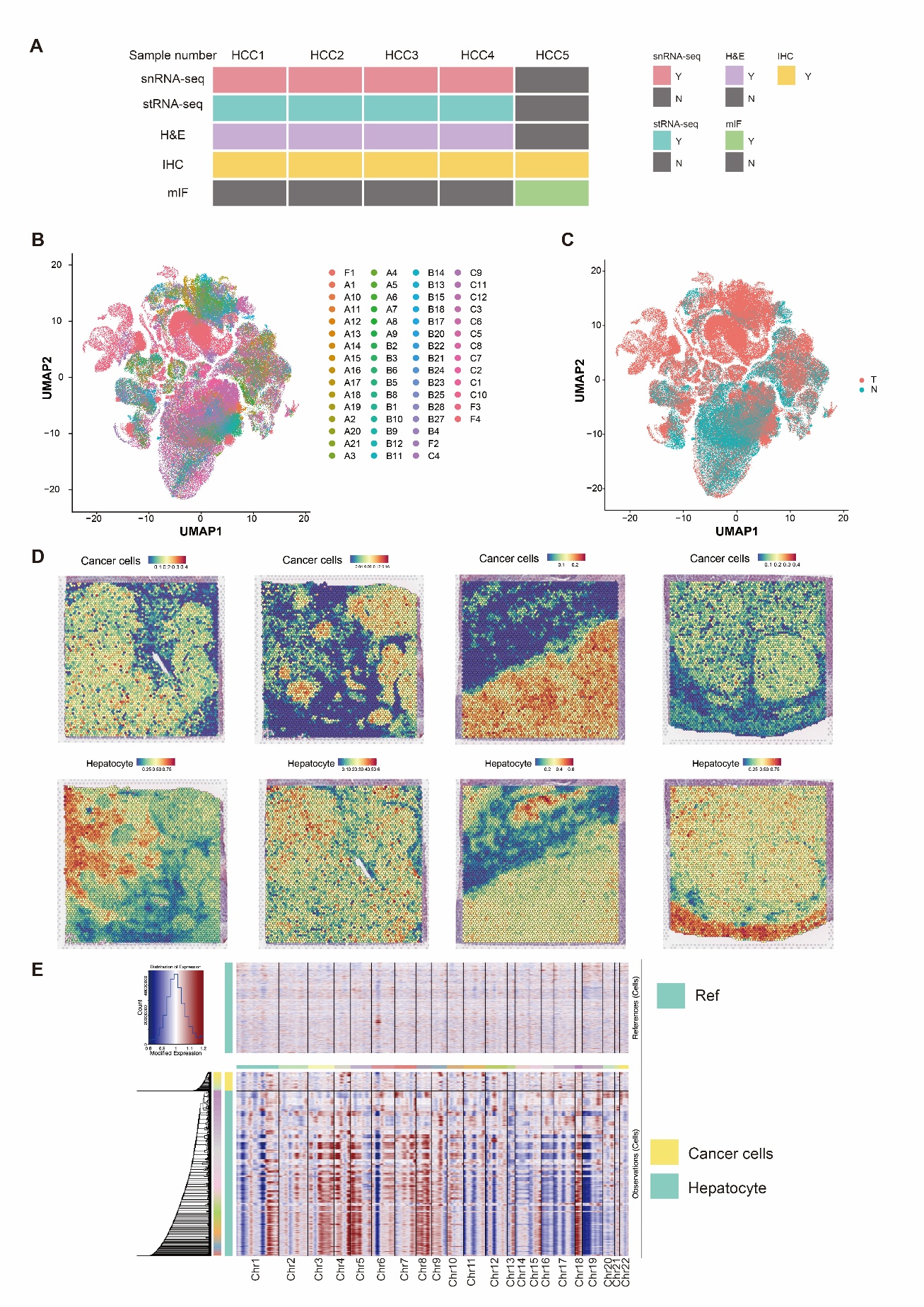
**

**Figure S1. Experimental details and the annotation process of stRNA-seq slides.** (A) Clinical characteristics and experimental details of cervical samples from HCC patient. (B) UMAP of stRNA-seq bins from 4 HCC samples. (C) UMAP of stRNA-seq bins from 4 HCC samples annotation results of T cells and N cells. (D) Expression of cancer cells and hepatocytes in four stRNA-seq slides. (E) Comparison of copy number variation (CNV) between cancer cells (82692 cells) and hepatocytes (3800 cells). The upper panel shows the CNV statuses for reference. The lower panel shows the CNV statuses of each cell from chromosome 1 to 22.


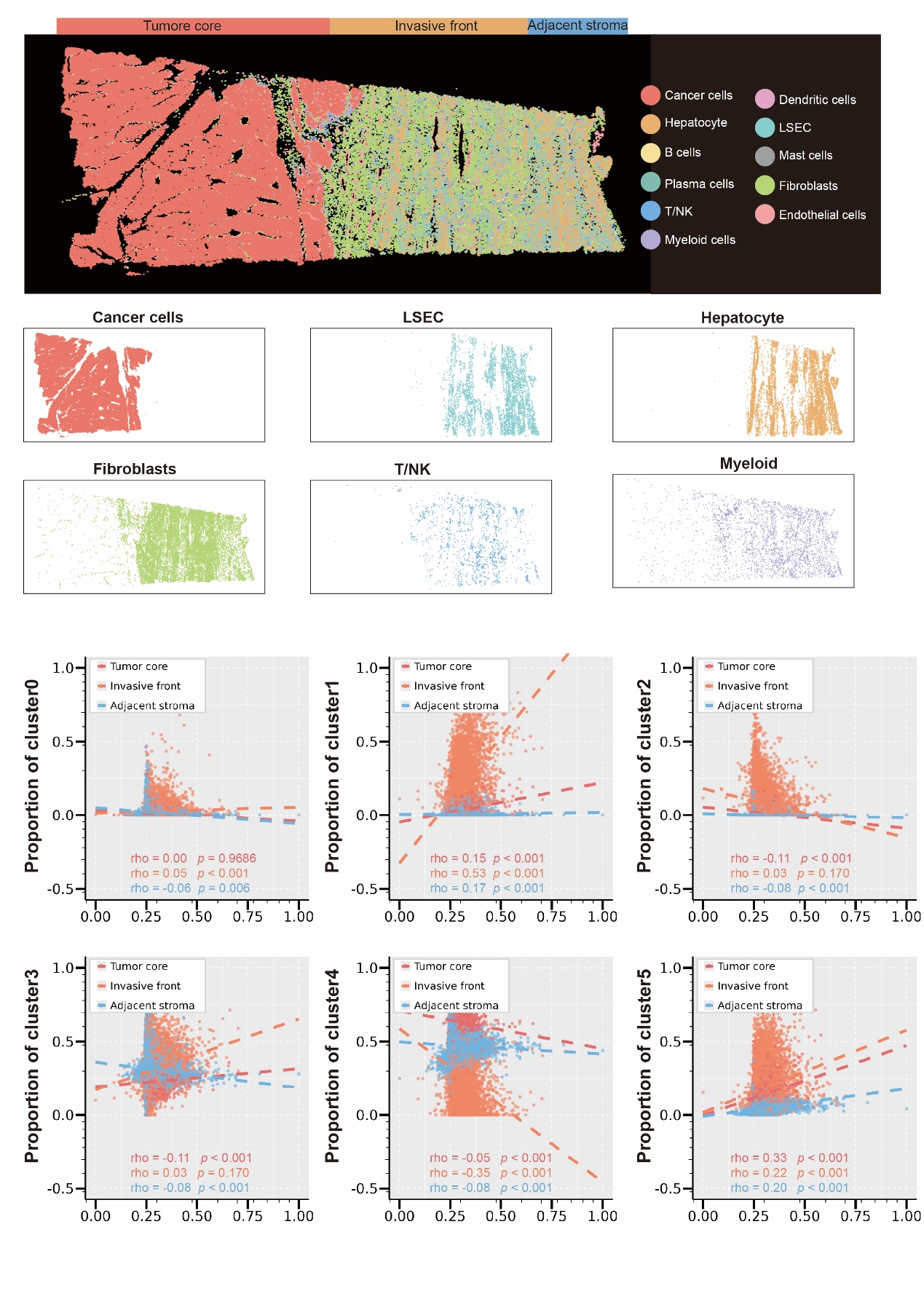
 **Figure S2. Validation the spatial distribution of cells by a set of HCC Xenium data.** Non-malignant cells such as LSECs, fibroblasts, myeloid cells, T_NK cells, and hepatocytes were significantly enriched in the adjacent stroma region.

**
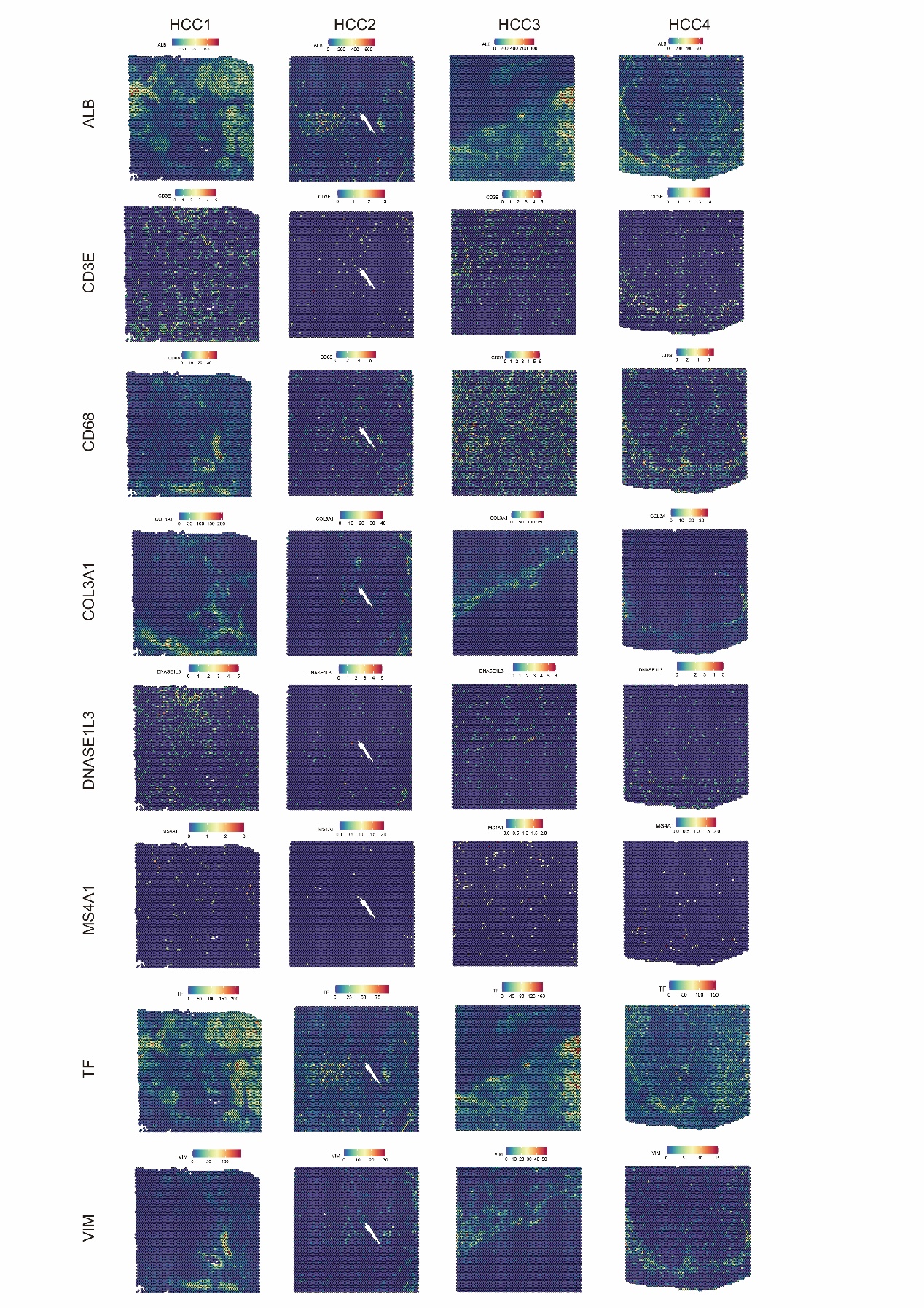
 Figure S3.** **Annotation results of the 4 stRNA-seq slides.** All samples were HCC samples. Eight genes were selected to represent different tissue types. Hepatocyte, ALB; T_NK cells, CD3E; Macrophage, CD68; Fibroblasts, COL3A1 and VIM; Endothelial cells, DNASE1L3; B cells, MS4A1; Cancer cells, TF.


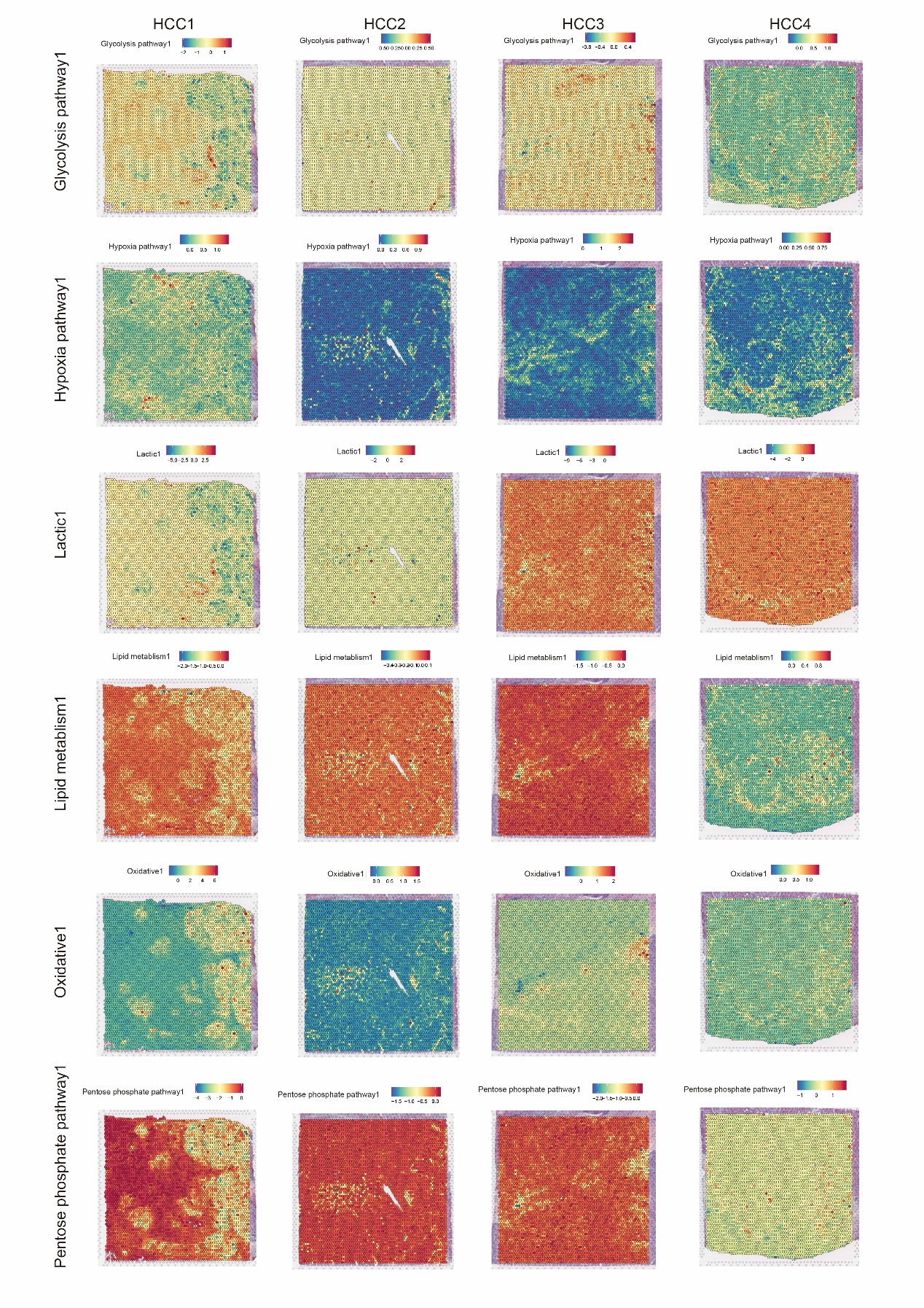


**Figure S4. Annotation results of the 4 stRNA-seq slides in six metabolic pathways.** All samples were HCC samples. The six metabolic pathways include: glycolysis pathway, hypoxia pathway, lactic pathway, lipid metabolism pathway, oxidative pathway and pentose phosphate pathway.


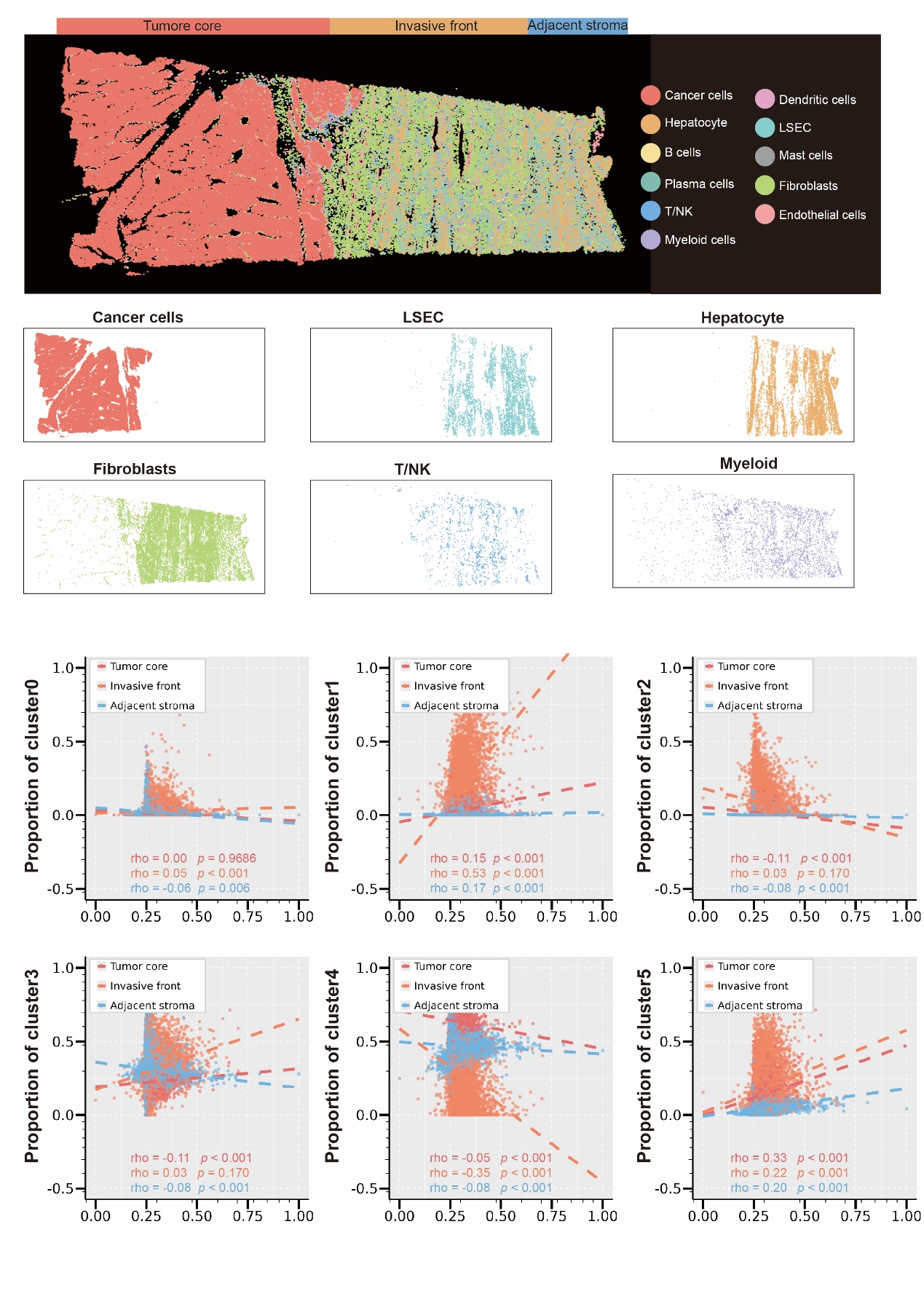


**Figure S5.** **Correlation analysis of the proportions of six fibroblast subtypes with hypoxia scores.** MyCAFs in the invasive front region of the tumor exhibited a significant positive correlation with hypoxic metabolism.


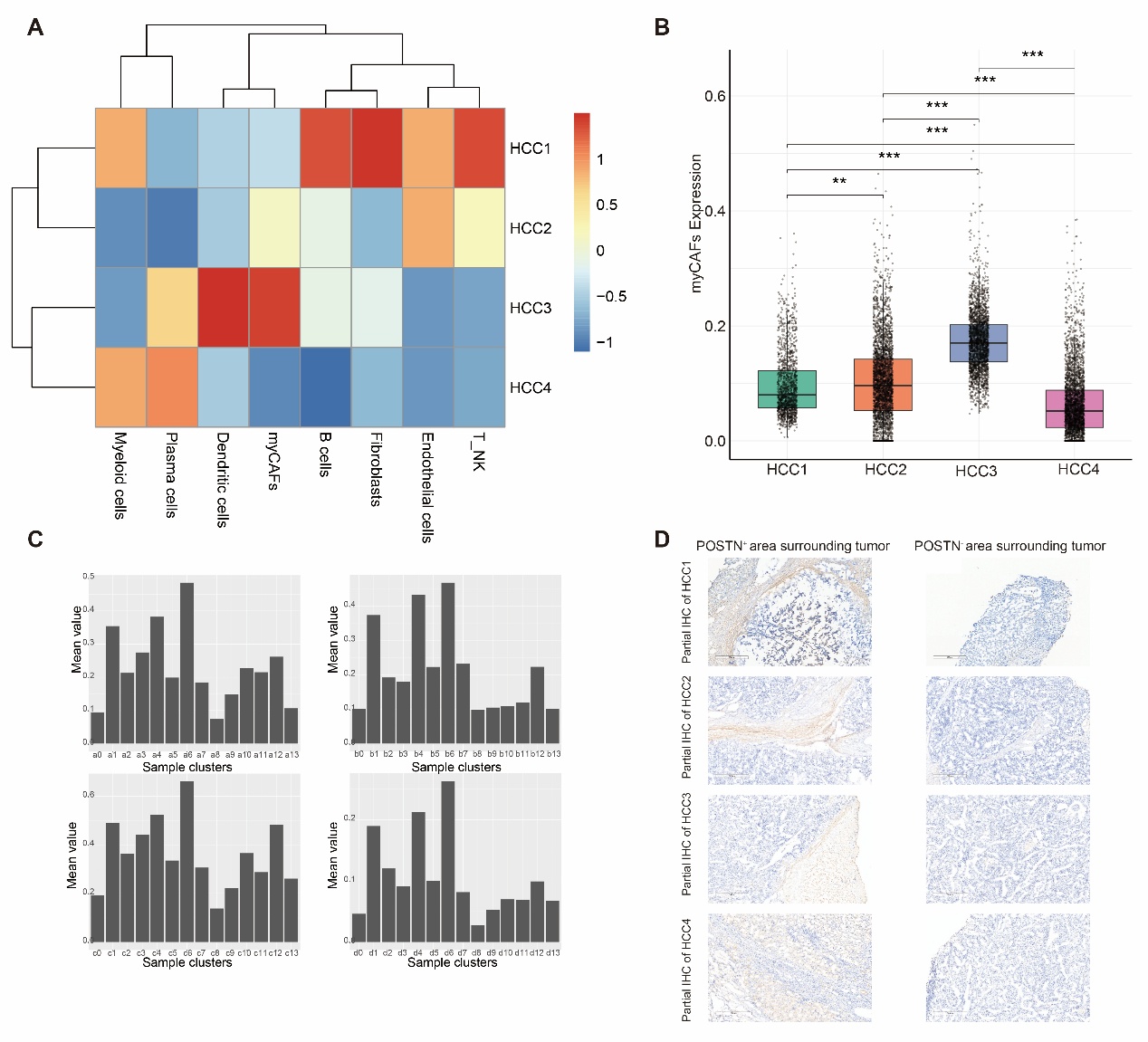


**Figure S6. Characterization of hmmyCAFs.** (A) Correlation between snRNA-seq cell types and samples. (B) Expression of hmmyCAFs in samples. (C) Expression of hmmyCAFs in sample clusters. (D) The IHC staining of POSTN in corresponding sections of HCC samples.


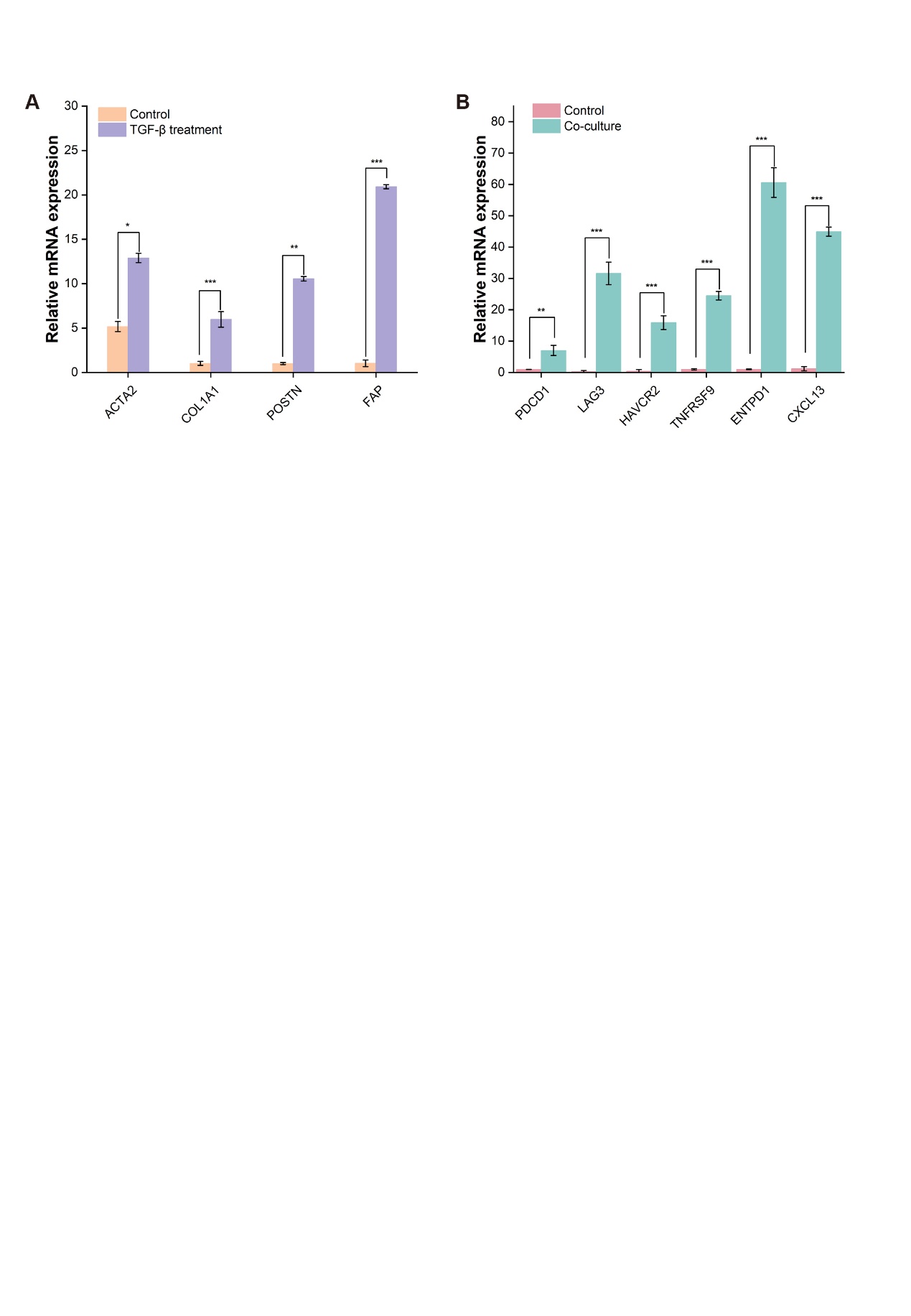


**Figure S7. Analysis of mRNA expression of hmmyCAFs and T Cell co-culture**. A) Analysis of mRNA expression levels related to hmmyCAFs phenotype. The relative mRNA expression levels of ACTA2, COL1A1, POSTN, and FAP genes in the control group (hepatic stellate cells, orange), and TGF-β-treated group (purple). B) Analysis of mRNA expression levels related to T cell exhaustion. The relative mRNA expression levels of PDCD1, LAG3, HAVCR2, TNFRSF9, ENTPD1, and CXCL13 genes in the control group (only T cells, pink), and the co-culture group T cells with hmmyCAFs (green). The *p* values were determined by Student’s t test: n.s., not significant; **p* < 0.05; ***p* < 0.01; ****p* < 0.001; *****p* < 0.0001.


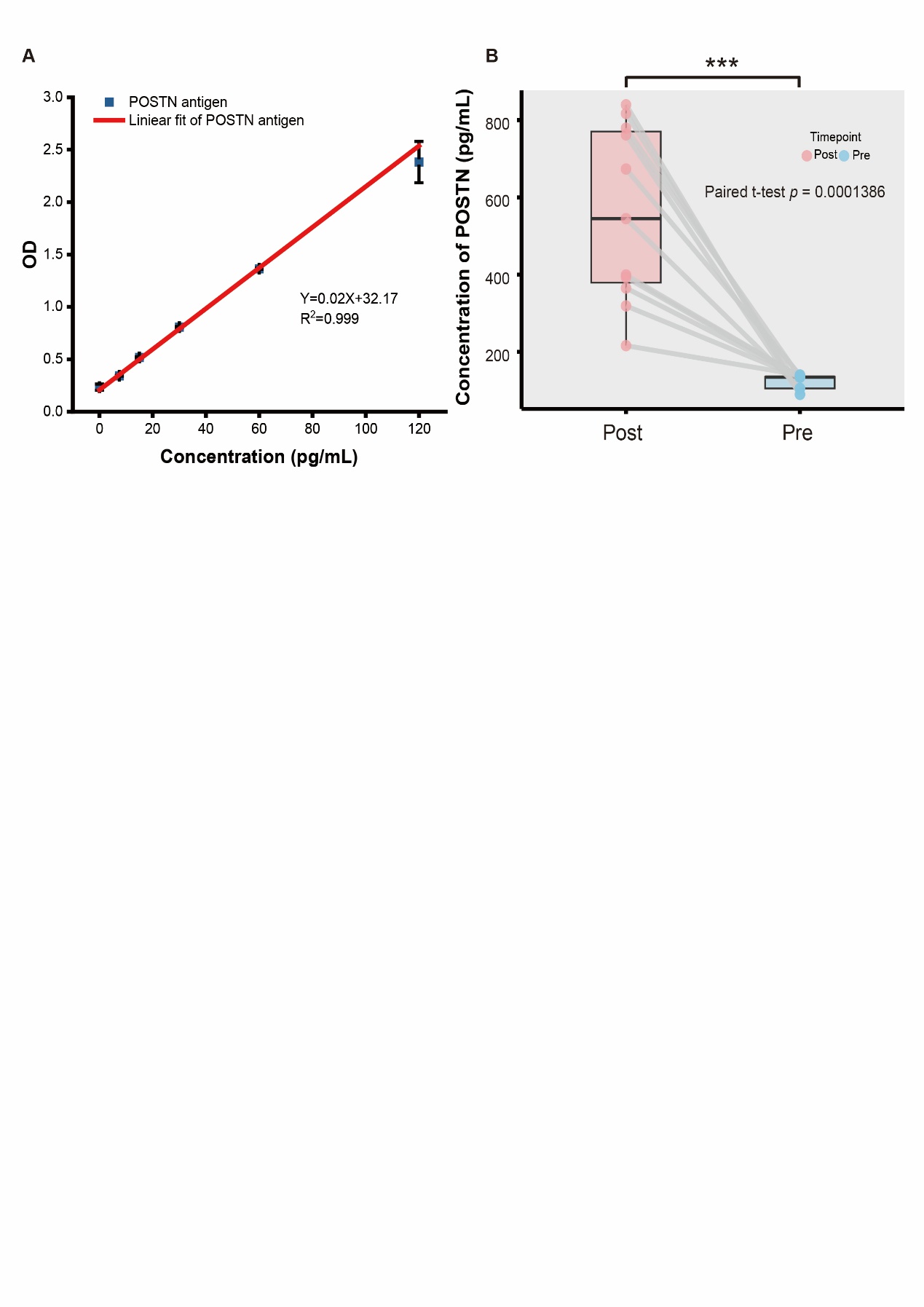


**Figure S8. Analysis of POSTN expression of before and after PD-1 immunotherapy HCC plasma samples**. A) Standard curve of POSTN antigen. The blue square represents the detection signal value of POSTN antigen, and the red solid line is the linear fitting curve. B) Paired comparison of POSTN concentrations in the same samples before (Pre) and after (Post) PD-1 immunotherapy. Pre, before PD-1 immunotherapy HCC plasma samples; Post, after PD-1 immunotherapy HCC plasma samples. The *p* values were determined by Student’s t test: n.s., not significant; **p* < 0.05; ***p* < 0.01; ****p* < 0.001; *****p* < 0.0001.
